# Assessment of Ammonia Concentrations and Climatic Conditions in Calf Housing Using Stationary and Mobile Sensors

**DOI:** 10.3390/ani14132001

**Published:** 2024-07-07

**Authors:** Julia Moser, Samuel Kohler, Jérémy Hentgen, Mireille Meylan, Gertraud Schüpbach-Regula

**Affiliations:** 1 School of Agricultural, Forest, and Food Sciences, Bern University of Applied Sciences, Länggasse 85, 3052 Zollikofen, Switzerland; samuel.kohler@bfh.ch (S.K.); jeremy.hentgen@bfh.ch (J.H.); 2 Department of Clinical Research and Veterinary Public Health, University of Bern, Schwarzenburgstrasse 155, 3097 Liebefeld, Switzerland; gertraud.schuepbach@unibe.ch; 3 Clinic for Ruminants, Vetsuisse Faculty, University of Bern, Bremgartenstrasse 109a, 3012 Bern, Switzerland; mireille.meylan@unibe.ch

**Keywords:** calf housing, livestock production, housing climate, management, noxious gases

## Abstract

**Simple Summary:**

Maintaining an appropriate housing climate has a major impact on the welfare of calves. The present study implemented a method for long-term measurement in the micro- and macroclimate range of calf housing. Observed differences between stationary and mobile measurements suggest that there are different microclimate areas in the housing. In particular, ammonia concentrations measured at microclimate level showed strong fluctuations and high peak values. The possibility of measuring ammonia in the direct environment of the calves might allow for further research on the relations between ammonia concentrations and animal health, and for investigations of management methods to optimize the housing climate at the animal level.

**Abstract:**

In calf fattening, housing climate conditions are essential for optimal performance and welfare. Validated methods to measure the long-term housing climate are lacking. The present study investigated climate parameters for 14 weeks in Swiss calf fattening housing with two different ammonia (NH_3_) sensors: six stationary sensors (Dräger Polytron 8100) were installed at animal level and four mobile sensors (Dräger x-AM 5100) were attached to the calves’ heads. Temperature, relative humidity, and carbon dioxide (CO_2_) concentrations were recorded by two stationary data loggers (testo 160 IAQ). Data were analyzed descriptively, and 4 h mean values of maximum NH_3_ concentrations of mobile and stationary sensors were compared using the Wilcoxon test for paired data. The 4 h mean values of temperature, relative humidity, and CO_2_ concentrations and the 4 h mean values of maximum NH_3_ concentrations of stationary and mobile sensors were analyzed by ANOVA in two linear models. The overall 4 h mean of maximum NH_3_ concentrations ranged between 5.9–9.4 ppm for measurements of stationary sensors and between 11.3–14.7 ppm for measurements of mobile sensors. The NH_3_ concentrations measured by mobile sensors showed significantly higher peak values and more fluctuations. Additionally, an interaction effect was observed between the NH_3_ concentrations measured by either sensor and CO_2_ concentrations (*p* < 0.01 (mobile sensors); *p* < 0.0001 (stationary sensors), temperature values (*p* < 0.0001 (both sensors)), and relative humidity (*p* < 0.0001 (both sensors)). The measurements of the implemented method showed that corresponding housing climate parameters fluctuated strongly, and NH_3_ reached high peak values. Validated measurement methods might allow for a detailed assessment of the housing climate in practice, and for further research on suitable management methods for housing climate optimization in the future.

## 1. Introduction

In recent years, precision livestock farming research has made substantial progress. New technologies allow for improved monitoring, decision-making, and management in livestock farming, thus contributing to enhanced animal health and welfare [1]. Sensors are an important element of this innovation. Accelerometers, for example, allow for early detection of lameness in dairy cattle. Similarly, activity monitoring of cows is also useful in detecting estrus [2].

In calf raising systems, the available technology to monitor calves’ health and welfare includes, for example, automatic milk feeding systems, early warning systems to detect increased coughing by sound [3], and accelerometers that monitor animals’ movement and behavior. Furthermore, infrared thermography for early detection of infections is already implemented [4]. While environmental sensors for measuring air quality in animal housing exist, calf-specific systems to monitor the microclimatic conditions in the barn are not yet available. Controlling climatic conditions in animal housing is considered to have a major impact on animal health and welfare. Monitoring climatic conditions by sensors provides a non-invasive approach to assess environmental conditions in calf pens. It provides real-time insight on air quality. This can inform farmers for decisions regarding ventilation and management measures, such as pen cleaning [4,5]. In calf fattening, this is of particular relevance because high disease and mortality rates are challenging. Poor animal health results in the widespread use of antibiotics in livestock farming and contributes to economic losses [6,7]. Environmental and management factors, such as climate and housing systems, are of major importance, especially for health in the early stages of life [8,9,10,11,12]. Previous studies showed associations of housing climate parameters, such as temperature and ammonia (NH_3_) concentration, with increased mortality and treatment incidences. Maintaining a good air quality was, thus, recommended, for instance, to keep NH_3_ concentrations low (<10 ppm) [11,12]. Based on previous studies, we assume, therefore, that good climatic conditions in the housing are of major importance for the optimal performance and welfare [13,14] of animals [9,12,15,16,17,18].

The main climate and environmental parameters described as being associated with animal health are ambient temperature, relative humidity, harmful gas concentrations, air contaminants (viruses and bacteria), dust, and air flow [9,12,17,18,19,20,21]. Noxious gases, such as NH_3_, damage and irritate the eyes and the respiratory tract at high concentrations. Recently it was shown that, in addition to other climate parameters, such as temperature and air velocity, prolonged exposure to NH_3_ concentrations above 4 ppm is associated with lung consolidation [12]. Indeed, calves are more sensitive to noxious gases, such as NH_3_, than adult cattle are [21,22]. Furthermore, poor air quality may increase exposure to airborne pathogens. The combination of high pathogen exposure and impairment of pulmonary defense mechanisms facilitates infection. Air quality is, therefore, considered to be of major significance as a predisposing factor in the pathogenesis of calf pneumonia [15,21,22,23].

In livestock facilities, NH_3_ is primarily set free by the breakdown of urea from the animals’ urine by the enzyme urease produced by microorganisms in their feces [24]. Consequently, as calf urine and feces are mixed in the housing environment, NH_3_ concentrations often reach harmful levels. Hence, the interior conditions play an important role in maintaining an animal-friendly housing climate [25,26]. Different management practices, such as the cleaning of the stable, ventilation, litter type, feed quality, and livestock density, as well as the structure of the housing, all influence air quality and the living conditions of the calves [20,26].

Empirical guidelines and recommendations exist both at the international and national levels. In relation to housing climate, the CIGR [27,28] published recommendations and guidelines for a fresh air supply and thermal conditions for calf housing. For animal housing, they recommend NH_3_ concentrations of a maximum value of 20 ppm and CO_2_ concentrations of no more than 3000 ppm. Recommendations from the Swiss Federal Government [20] regarding housing climate are available in Switzerland as well, where relevant parameters for the assessment of housing climate in cattle housing are described. The Swiss Federal Government recommends optimal and threshold values for temperature (between 5 and 20 °C), relative humidity (between 50 and 80%), NH_3_ concentration (maximum 10 ppm), and CO_2_ concentration (maximum 3000 ppm) [20]. Furthermore, possible measurement methods for the individual climate parameters are described. However, it is difficult to verify the implementation of these guidelines and recommendations in practice. A variety of commercially available devices for the measurement of individual climate parameters exists. To date, measurement devices for practical use have been tested or validated in only a few studies. Therefore, evidence is sparse, especially for measurement devices for noxious gases, such as NH_3_. In comparison, measuring devices for long-term monitoring of housing temperature and relative humidity have already been used in previous studies for housing climate measurements [29,30,31]. A recent study analyzed repeated point measurements of temperature, relative humidity, carbon dioxide (CO_2_), and NH_3_ concentrations, as well as continuous measurements of temperature and relative humidity over 72 h in veal calf housing [32]. They stated that results of point measurements are highly variable, particularly for noxious gases, and, thus, are difficult to interpret, and they did not observe significant associations between the results of measurements of climate parameters and animal health indicators. However, continuous measurements seemed to be more appropriate to assess the housing climate, because strong fluctuations and deviations from optimum values were observed by continuous measurements, but not by point measurements. Continuous measurements of noxious gas concentrations were not performed in this study, but point measurements showed a large variability in the values of NH_3_ and carbon dioxide (CO_2_) concentrations measured at the same time in different locations in the housing [32].

In the past, climate parameters in animal facilities were assessed by diverse measurement methods. Concentrations of total NH_3_, CO_2_, methane, and other emissions have been assessed in housing in several studies [33,34,35,36]. Nevertheless, there is no established standard method, and commonly applied methods differ in cost, complexity, sensitivity, and precision [37]. In practice, standardized methods for evaluating and regulating animal friendly housing climates are lacking [38]. To assess the actual NH_3_ exposure of calves under practice conditions, a measurement method is required that closely reflects the true NH_3_ concentration they encounter.

Multipoint sampling is useful to accurately estimate the exposure of animals to a certain climate parameter, such as NH_3_. In order to obtain the best possible resolution and coverage of the environment to be sampled, climate parameters are measured in several locations [37]. In contrast, single-point measurements, for example with one single stationary device, have often been applied in previous studies to assess the exposure of animals. Furthermore, researchers often used short-term or one-time measurements, or one-time measurements at several sampling spots, such as with portable devices [12,29,32]. One-time measurements only represent the current conditions at the sampling location and/or time and are, thus, not a reliable indicator of the development of NH_3_ concentrations or other housing climate parameters over time [37]. It is known that NH_3_ concentrations, temperature, and relative humidity may show diurnal, seasonal, and long-term variations. Therefore, long-term monitoring of climate parameters in animal housing, is essential for accurately assessing housing climate conditions [21,32,39,40].

In previous studies, measurement devices have been mounted on the housing ceiling or outside the animal compartments, rather than on the animals or in their immediate surroundings [12,32,41,42]. Such approaches only cover the macroclimate area of the housing. However, macroclimate measurement values do not necessarily reflect the microenvironment. Measurements in the animal compartments itself and at animal level, reflect the microclimate, and, therefore, the climatic conditions in which the animals actually live. The position of the measuring device has been shown to be important for obtaining relevant measurement values. Since NH_3_ is produced in the microclimate range, it should also be measured there. Likewise, measurements should be taken in the immediate environment of the animals. In fact, it is assumed that the assessment of the conditions inside the housing at animal level is essential, especially for developing future regulation systems to maintain an animal-friendly housing climate [26].

The aim of the present study was to collect reliable data on housing climate in the microclimate range. This study involved long-term data collection of macro- and microclimatic conditions in calf housing. Therefore, a method to measure NH_3_ concentrations in the direct environment of the calves was implemented. Measurements performed by stationary and mobile devices were compared, and the applicability of the devices under practice conditions was evaluated. We hypothesized that NH_3_ concentrations measured with the mobile NH_3_ sensors attached to the calves’ heads would be significantly different from the measurements with stationary devices. Furthermore, we expected variations in temperature, relative humidity, and CO_2_ concentrations to correlate with NH_3_ concentration fluctuations. By performing measurements in the animal’s direct environment, we aimed to provide important information on the air quality and microclimate within animal housings.

## 2. Materials and Methods

### 2.1. Housing and Ventilation

**Figure 1:**
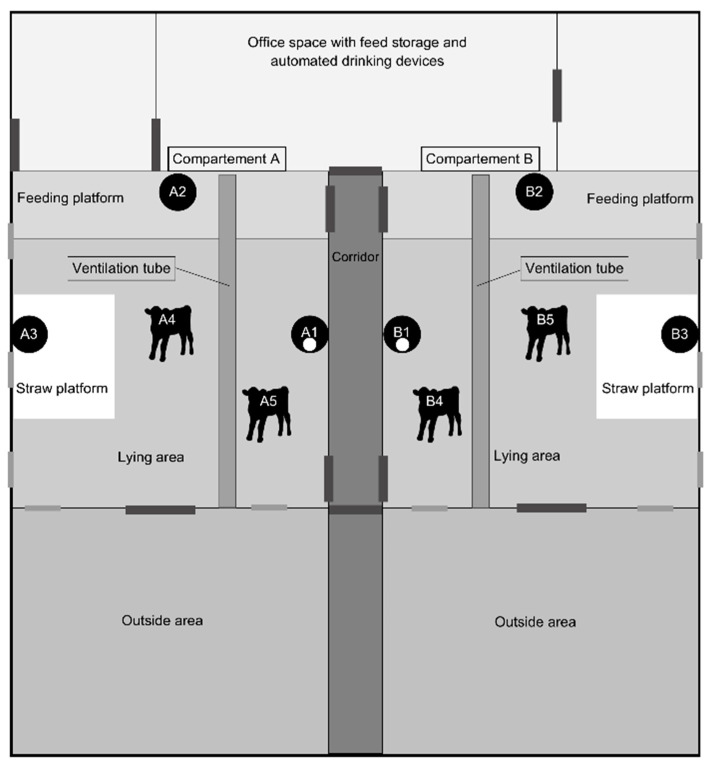
Schematic representation of the experimental barn and experimental set-up, with stationary NH_3_ measuring devices (A1–A3, B1–B3) and mobile NH_3_ measuring devices (A4, A5, B4, B5). The white dots at position A1/B1 represent the Testo data loggers for measurements of the temperature, relative humidity, and CO_2_ concentration. The black bars represent doors and gates. The gray bars represent windows. The different areas are indicated by different shades and are named accordingly.

The study was conducted in Switzerland in an experimental calf fattening barn in Hendschiken (Canton Aargau). The measurements lasted for two months, corresponding to one fattening period, from 5 June to 8 August 2019. The barn is a free-standing building at 473 m.a.s.l. (Google Maps, 2021) [43]. The building envelope is made of concrete and wood. It is a single barn with two compartments of equal size, A and B. A paved corridor divides the two compartments in the center of the barn. A half-height fence made of metal bars with plastic panels separates the compartments from the corridor. Each compartment, therefore, has such a fence adjacent to the corridor which runs through the middle of the barn. There are five windows per compartment (two windows located to the east, left and right of the gate to the outside area, with the other three on the outer wall of each compartment; see Figure 1).

Each compartment consists of a feeding platform oriented to the west with automat-ed drinking devices (4 Förster drinkers per compartment, 9.25 calves per suckling station). A lying area littered with deep straw (73.5 m^2^, 1.89 m^2^ per animal) is adjacent to the feeding platform, and each compartment has a permanently accessible outside pen (69 m^2^, 1.87 m^2^ per animal). The unroofed paved outdoor area is oriented to the east. One gate per compartment leads to the outdoor area, and plastic curtains in the outlet gates reduce the air draft.

Two ventilation tubes (VET.SMART, NEOWOLF GmbH, Graz, Austria) are set in the barn, one above each compartment. Each tube is 10.6 m long with a diameter of 40.93 cm, with holes at regular intervals (outer tube: 5 lines with 30 holes per line; distance between holes in the same line: 33.2 cm), and a fan (Multifan, Vostermans Ventilation, Venlo, The Netherlands) with a diameter of 362 mm and a delivery volume of 2940 m^3^/h at 37 Pa static pressure. The fan is installed on the outer side of the barn, above the outside area. The ventilation system is designed in such a way that the fresh air arrives at the calves’ lying area at a maximum speed of 0.3 m/s. The ventilation was switched on constantly during the whole experiment. To avoid heat stress, farm personnel removed the barn windows and opened the gates as necessary. Throughout the experiment, we documented any changes to windows, doors, and ventilation. The farm personnel documented manure removal, litter consumption, and ventilation parameters on a specially designed form during the entire measurement period.

### 2.2. Animals

During data collection, a total of 74 calves was present in the barn. Calves were kept according to the guidelines of Coop Naturafarm Kalb [44]. The purchased calves underwent general examination carried out and documented by the farm personnel upon arrival. This general examination is carried out routinely for each new fattening cycle on that particular farm. After arrival, the calves were uniformly distributed between the 2 barn compartments, into groups of 37 calves each. All calves arrived at the farm within two days. Group A consisted of 21 female and 16 male calves, and group B consisted of 20 female and 17 male calves. They belonged to common dairy, beef, and mixed breeds (e.g., Holstein Friesian, Braunvieh, and Limousin, respectively). The calves originated from 70 different birth farms and were between 21 and 83 days old when they were brought in for fattening. The farm personnel weighed each calf upon arrival (57–89.5 kg), three times during the fattening period after 21, 56, and 83 days (13.06.19, 18.07.19, 14.08.19), and on the day of slaughter (first calf slaughtered 09.08.19), and the weights were recorded in the barn journal. Additionally, the farm personnel kept records of calf health over the entire fattening period, where they registered the health status (incl. daily rectal temperature of sick calves) and any medication used. The calves left between 121 and 171 days of age, with slaughter weights between 160 and 247.5 kg.

### 2.3. Sensors

The following sensors were used to carry out measurements of climate parameters.

#### 2.3.1. Stationary Device for NH_3_ Measurement

A Dräger Polytron 8100 data logger with DS NH_3_ FL Agriculture electrochemical sensor (Drägerwerk AG & Co. KGaA Dräger. 2023, Lübeck, Germany) was used to measure NH_3_ concentration. According to the manufacturer’s information, the sensor is capable of measuring NH_3_ concentrations in a range of 0–300 ppm, with a precision of 1 ppm, over a temperature range of −40–65 °C. For detailed information on sensor technology refer to [45,46,47] and see supplementary material.

#### 2.3.2. Mobile Device for NH_3_ Measurement

A Dräger x-Am 5100 data logger with Prototype NH_3_ electrochemical sensor (Drägerwerk AG & Co. KGaA Dräger. 2023) was used to measure NH_3_ concentration. According to the manufacturer’s information, the sensor is capable of measuring NH_3_ concentrations in a range of 0–100 ppm, with a precision of 1 ppm, over a temperature range of −40–65 °C.

#### 2.3.3. Stationary Device for Temperature, Relative Humidity, Atmospheric Pressure, and CO_2_ Measurement

A Testo 160 IAQ (Testo SE & Co. KGaA Testo AG. 2003, Titisee-Neustadt, Germany) radio data logger was used to measure the temperature, relative humidity, atmospheric pressure, and CO_2_ concentration. According to the manufacturer’s information, this sensor is capable of measuring temperature in a range of 0–50 °C, with a precision of ±0.5 °C; relative humidity, range 0–100% rF, with a precision of ±2.0%; absolute pressure, range 600–11,000 mbar, with a precision of ±3 mbar; CO_2_, a concentration range 0–5000 ppm, and a precision of ±50 ppm.

### 2.4. Set-Up Measuring Devices

#### 2.4.1. NH_3_ Sensors

We installed a total of six stationary NH_3_ sensors (A1–A3, B1–B3) and four mobile NH_3_ sensors (A4, A5, B4, B5) in the barn (see the barn plan in Figure 1 for positions). The stationary sensors were equally distributed between compartments A and B. They were installed approximately 25 cm above the floor, i.e., at the level of the calves’ head when they were lying on the litter mat. Metal wire cages of 25 × 20 cm × 20 cm served as protection for the stationary NH_3_ sensors. The mobile sensors were prototypes made to perform measurements in the nose area of the calves. For this purpose, we randomly chose two calves per compartment to be fitted with a mobile NH_3_ sensor attached to their head with a halter. The halter maintained the sensor in position, approximately 10 cm behind the nose. The calves equipped with a mobile sensor were able to move freely in their compartments and the outdoor area, like all other calves of the group.

The measurement intervals for all NH_3_ sensors were set to 1 s, and the maximum value during each 10 min interval was recorded. A recording of the peak values was chosen to potentially determine the frequency and duration of the exposure to NH_3_ values that exceeded the recommended limits. We checked all of the sensors every three weeks. For this purpose, the latest versions of the software provided by the manufacturer were used: Dräger PolySoft (Dräger Safety AG & Co. KGaA. 2021, Lübeck, Germany) and Dräger cc-Vision (Dräger Safety AG & Co. KGaA. 2021, Lübeck, Germany). Furthermore, we replaced the dust filters and calibrated the sensors with a calibration gas from the manufacturer every three weeks. The calibration gas had an NH_3_ concentration of 50 ppm (+/−5%). To avoid data loss due to premature switch-off, we replaced the batteries of the mobile NH_3_ sensors once a week. The stationary sensors were powered by electricity.

#### 2.4.2. Temperature, Relative Humidity, Atmospheric Pressure, and CO_2_ Sensors

One Testo IAQ 160 radio data logger was installed in each compartment (A and B) in the same locations as the stationary NH_3_ sensors, at positions A1 and B1 (see Figure 1). The recording interval was set to 10 min and instantaneous values were recorded. Calibration and battery replacement during the measurement period was not needed for these data loggers. The data loggers automatically stored the measurement data in a data cloud, where they could be accessed at any time by the study team.

### 2.5. Statistical Analysis

Data from NH_3_ sensors were available as text files and were converted to the Excel (Microsoft Corporation Microsoft Excel 2019) format for further processing. Implausible data, i.e., measured values outside the measurable range of the devices, were registered as missing data in the data set. Measurement values registered during times at which the measurement devices were calibrated were excluded from the dataset, because calibration influenced the measured values, and, thus, resulted in incomplete measures. For the exclusion of data, the last timestamp with a measurement value before and after calibrating served as reference points. We subtracted 30 min from the last measurement value of the measurement device that was calibrated first. This determined timestamp served as the beginning of data exclusion. The first measurement value of the last calibrated measuring device served as the end point, to which 30 min were added, and data were removed up to the determined timestamp. All values recorded during the resulting timespan were removed from the data set for all measuring devices (NH_3_ concentration, CO_2_ concentration, temperature, and relative humidity). This procedure was repeated for all calibrations and replacements of batteries. In addition, values of continuous measurements were not obtained at certain times, due to the failure of the measurement devices. An overview of the number of recorded values available for descriptive and statistical analyses is given in Table 1, Table 2, Table 3 and Table 4.

The statistical analyses were carried out using the statistical program R (R Core Team R: A Language and Environment for Statistical Computing 2021). We calculated 4 h mean values of the recorded barn climate parameters individually for the respective devices. This resulted in 4 h means of the recorded peak values of NH_3_ concentrations (stationary NH_3_, mobile NH_3_) and 4 h means of the recorded point values of temperature, relative humidity, and CO_2_ (Testo data logger). First, the data were visualized and analyzed for a normal distribution, and a paired *t*-test was performed. Because the data were non-normally distributed and not independent, we used a Wilcoxon test for paired data to compare the peak values of NH_3_ measurements from mobile devices with stationary devices. For this purpose, the previously generated 4 h mean peak values of all stationary NH_3_ sensors were combined to one mean peak value per 4 h. The same was carried out for all mobile NH_3_ sensors. This resulted in overall 4 h mean peak values of NH_3_ concentrations per sensor type.

To test for effects between peak NH_3_ concentrations and the other measured climate parameters, we conducted a two-way analysis of variance (ANOVA) on two linear models. For this purpose, the overall 4 h mean peak values of NH_3_ concentrations measured with stationary and mobile devices were used as response variables, and overall 4 h mean values of temperature, relative humidity, and CO_2_ concentration were used as explaining variables. To achieve better fit and an approximate normal distribution, data were log-transformed.

## 3. Results

### 3.1. NH_3_ Concentrations

Table 1 shows descriptive statistics of the NH_3_ concentrations measured by each sensor over the entire study period. Because of a technical defect, mobile sensor B4 did not generate data between 24 and 31 July, and after 3 August 2019. No other measurement gaps occurred; the other sensors recorded data continuously. Overall, the 4 h mean values of maximal NH_3_ concentrations measured by the mobile sensors (A4–A5, B4–B5) were slightly higher than the 4 h mean values of the maximal NH_3_ concentrations measured by the stationary sensors (A1–A3, B1–B3, Table 1, Figure 1) (*p* < 0.001). The overall means of the NH_3_ concentrations measured by mobile sensors were 10 ppm, whereby a large proportion (47.50–72.13%) of individual measured maximal NH_3_ concentrations were above the recommended limit of 10 ppm [20]. The overall means of the NH_3_ concentrations measured by stationary sensors were below 10 ppm, whereby the proportion (20.58–36.72%) of individual measured maximal NH_3_ concentrations above the recommended limit of 10 ppm was smaller than for NH_3_ concentrations measured by mobile sensors. The proportion of measured NH_3_ concentrations below 5 ppm was lower for the mobile sensors, compared to the stationary sensors (5.89–20.11%, 24.27–59.35%).

**Figure 2:**
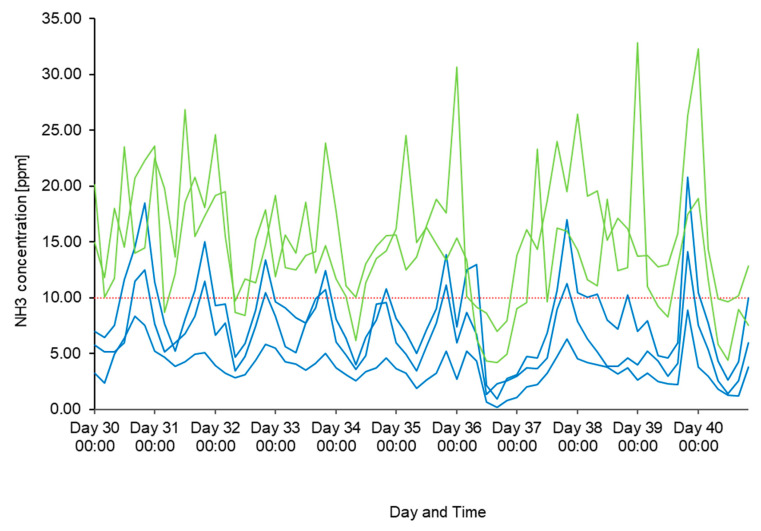
Four-hour mean values of NH_3_ concentrations in compartment A for mobile and stationary measuring devices over the course of ten days. Light green lines = mobile sensors A4 and A5, blue lines = stationary sensors A1 to A3, red dashed line = 10 ppm (recommended maximal NH_3_ value for calf barns). Indicated are the 24 h time intervals from day 30 to day 40.

Figure 2 shows an example of the calculated 4 h mean values of NH_3_ concentrations over a course of 10 days. The NH_3_ concentrations fluctuate strongly throughout the day, and diurnal patterns are visible. NH_3_ values recorded by mobile sensors were slightly higher, showed higher peak values and stronger fluctuations compared to the NH_3_ values measured by stationary sensors.

**Figure 3:**
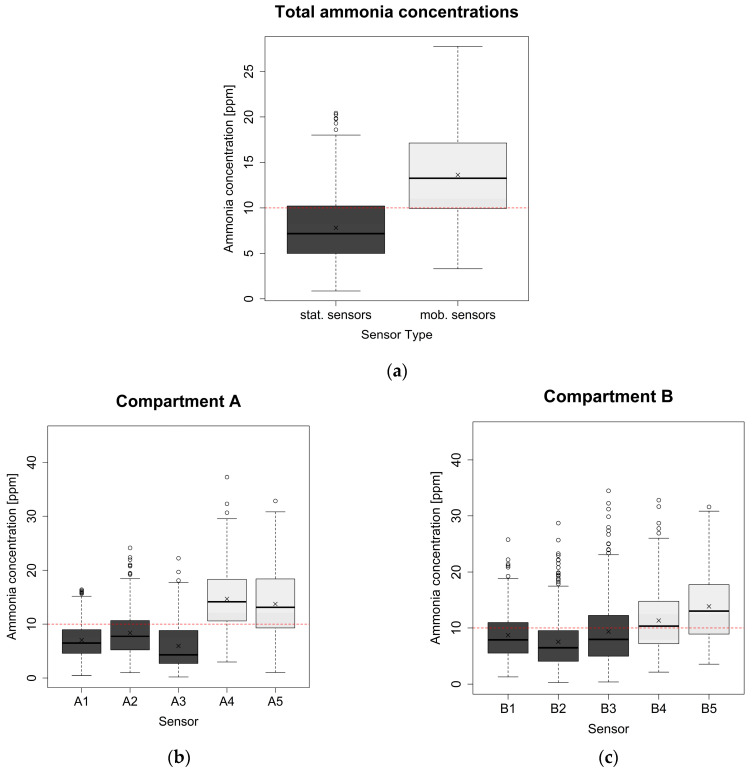
(**a**) The boxplots show all NH_3_ concentrations measured by stationary and mobile sensors over the entire study period in both compartments of the barn for stationary and mobile measurement devices separately (*p* < 0.001). (**b**) The boxplots show NH_3_ concentrations of each measuring device in compartment A (A1–A5) over the entire measurement period. (**c**) The boxplots show NH_3_ concentrations of each measuring device in compartment B (B1–B5) over the entire study period. Dark grey boxes = stationary NH_3_ measurement devices; light grey boxes = mobile NH_3_ measurement devices.

The boxplots in Figure 3 show the data distribution in the NH_3_ measurements per-formed with stationary and mobile devices. Measurements from mobile devices are distributed in a higher range compared to the measurements from the stationary devices. This is visible in the overall representation of measurements across all stationary and all mobile devices over the entire study period (Figure 3a), as well as in the boxplots for the individual devices and compartments (Figure 3b,c). The overall 4 h mean peak values of NH_3_ concentrations measured by stationary and by mobile devices were analyzed using the Wilcoxon test for paired data, and a statistically significant difference was found (W(378) = 23, *p* < 0.001).

### 3.2. CO_2_ Concentrations, Temperature, and Relative Humidity

Table 2, Table 3 and Table 4 show an overview of the climate data measured by the two Testo IAQ 160 climate sensors over the entire study period. After 10 July 2019, humidity values recorded by the Testo B1 humidity sensor were permanently very high (above 90% relative humidity). Because of the high humidity over a long time, the humidity sensor corroded. The high values occurred after the barn was cleaned out on 11 July 2019. We suspect that the sensor was contaminated with splash water during this procedure. Data on relative humidity from this sensor were, therefore, classified as implausible after 10 July 2019, and were excluded from further analyses. There were slightly more data outages during measurements with Testo IAQ 160 devices (29 days), compared to the NH_3_ measurements (14 days). Nevertheless, plausible data could also be generated over a long time period for these three climate parameters. CO_2_ concentrations were generally below the recommended maximum, and only a small percentage were above 3000 ppm (Table 2). In contrast, the temperature exceeded the recommended limit of 20 °C most of the time during the summer measurement period, and very high values were recorded, with maximum temperatures of up to 34 °C (Table 3). Finally, relative humidity in the experimental barn was mostly within the recommended limits of 50–80%; however, values above and below the recommended values were observed as well (Table 4).

**Figure 4:**
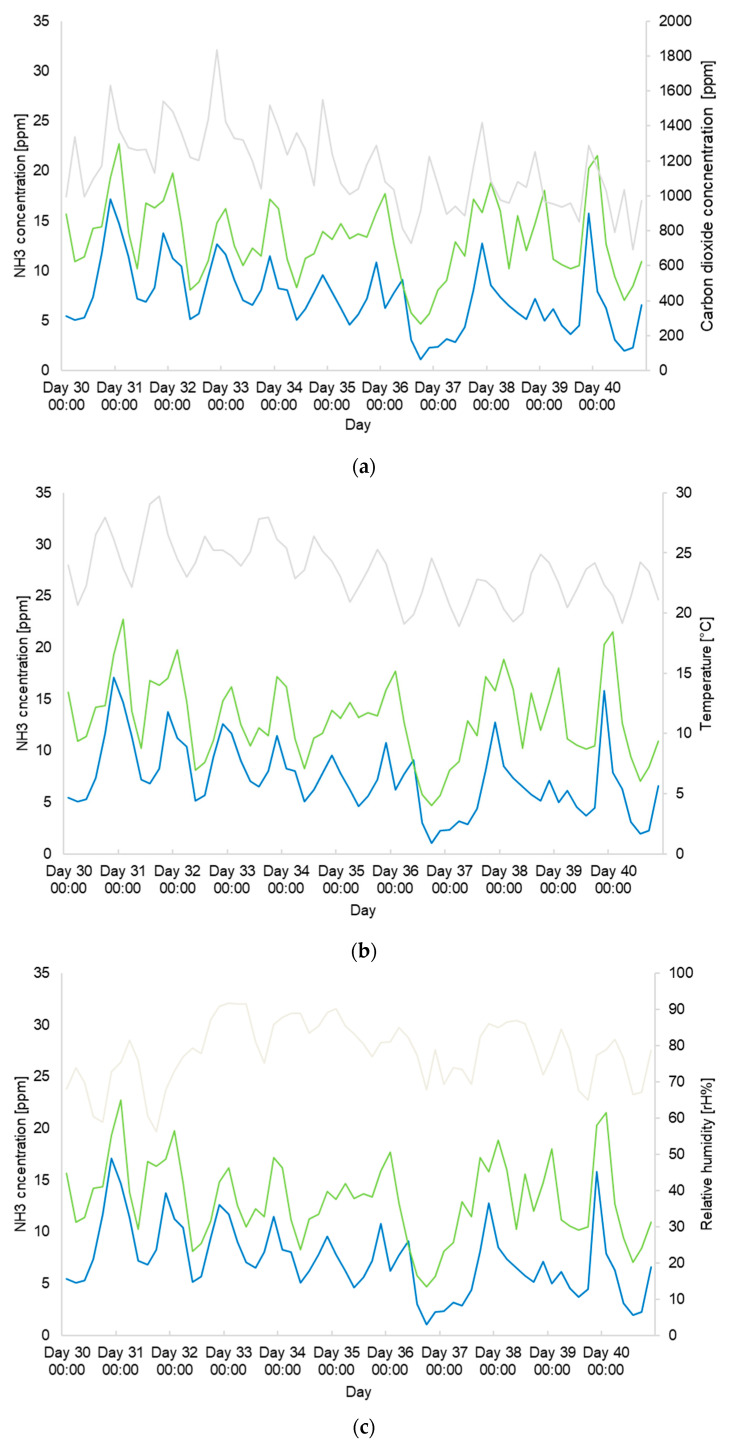
The NH_3_ concentrations measured with the mobile and stationary devices are depicted over the entire study period from 5 June to 8 August 2019, with (**a**) the measured CO_2_ concentrations, (**b**) the measured temperature values, and (**c**) the relative humidity (after 10 July, only values from Testo A1 due to a technical defect in Testo B1). The NH_3_ concentrations of the mobile devices (A4, A5, B4, B5) were combined into a 4 h average and are shown in light green. The NH_3_ concentrations of the stationary devices (A1–A3, B1–B3) were combined into a 4 h average and are shown in blue. The other climate parameters measured with the Testo (A1, B1) were summarized to a 4 h mean value and are shown in gray. Indicated are the 24 h time intervals from day 30 to day 40.

All three climate parameters, namely CO_2_ concentration, temperature, and relative humidity, showed interaction effects with NH_3_ concentrations. The ANOVA analysis revealed an interaction effect between the three climate parameters with NH_3_ concentrations measured by mobile as well as stationary devices. A significant effect was observed between the NH_3_ concentrations measured by the mobile sensors with the measured CO_2_ concentrations (F(375, 1) = 7.9, MSE = 0.16, *p* < 0.01), temperature values (F(375, 1) = 167.97, MSE = 3.4, *p* < 0.0001), and relative humidity (F(375, 1) = 77.15, MSE = 1.57, *p* < 0.0001) (Figure 4). For the stationary NH_3_ measurement devices, CO_2_ concentration (F(375, 1) = 55.19, MSE = 1.45, *p* <0.0001), temperature (F(375, 1) = 180.13.5, MSE = 4.73, *p* < 0.0001), and relative humidity (F(375, 1) = 156.75 MSE = 4.11, *p* < 0.0001) also showed significant effects on the NH_3_ concentration (Figure 4).

## 4. Discussion

A new long-term measurement method for NH_3_ concentrations in micro- and macro-climatic conditions was successfully implemented in an experimental calf barn in Switzerland. To our knowledge, this is the first study where NH_3_ concentrations were continuously measured in the barn microclimate with mobile sensors attached directly to the calves’ heads, combined with continuous stationary measurements in the macroclimatic range. The continuous measurements provided information about typical NH_3_ concentrations in a barn in summer. The devices performed well under real-world farm settings. Thus, data on NH_3_ concentrations collected in the barn micro- and macroclimate range, as well as on CO_2_ concentrations, relative humidity, and temperature in the barn macroclimate, were obtained over several months in the direct surroundings of fattening calves.

To properly assess a barn’s climate, it is important to consider conditions at the microclimatic scale [26,39,48]. By performing NH_3_ concentration measurements with sensors attached to the calves’ heads, we first found a significant difference between measurements taken by stationary sensors and measurements taken by mobile sensors. Second, we found high variance between measurements not only in the data from mobile devices, but also for stationary devices. Therefore, we confirm the results from previous studies, where strong fluctuations in the climate of livestock and cattle barns were recorded as well. Depending on housing system, strong seasonal but also diurnal effects were observed, especially in cattle and calf housing, which were characterized by wider variations in barn climate [16,39,40]. There is evidence for the existence of microenvironments in calf barns. In a previous study it was observed that, in terms of temperature, pens may represent different microenvironments within a barn [16]. In our study, we could show that such microenvironments exist in terms of temperature, as well as for noxious gases.

Placement of the measurement devices is an important factor to accurately assess barn climate. NH_3_ concentrations decrease with increasing distance from the source location [26]. In the study by [26], measuring devices were installed at different distances above the floor, and it was found that NH_3_ concentrations decreased with increasing height in the barn. Consequently, it is important to install the measuring devices at animal level; otherwise, the barn climate might be misjudged. Therefore, in our study, we installed all measurement devices at the level of the animals’ noses, and our findings are in agreement with those of the study mentioned above. The NH_3_ concentrations measured by the mobile devices vary more significantly than measurements from stationary sensors. Because calves moved freely around the barn, the measurements originate from different locations and heights. However, variations were also observed in the measurements of the stationary sensors, which were installed at different locations as well. Since barn climate is variable and comes with the expectation of strong spatial fluctuations, previous studies already found that multipoint sampling and a high spatial resolution of measurement points is important [37,38]. By performing point measurements, or measurements over short time periods, the fluctuation and variability (spatial and temporal) of NH_3_ concentrations observed in this study likely would have gone undetected. Furthermore, our results were also in line with the findings of [48], who detected different accumulation areas, with higher gas concentrations in the microclimate range. They observed high spatial variation in noxious gases in dairy buildings. Spatial distribution of noxious gases is not uniform and is influenced by many complex factors, such as air temperature, air flow, which is affected by ventilation, building structures, and animal distribution in the building [37]. On this basis, one could expect that, depending on a calf’s location in the barn, the NH_3_ concentrations might vary strongly. Our measurement method covered this range, by allowing calves equipped with mobile sensors to range freely in the inside and outside areas. If only stationary sensors had been used for barn climate assessment, those fluctuations and sudden high peaks, which might be caused by such accumulation areas in the microclimate, would have been lost.

Continuous multipoint sampling, as performed in this study, can provide more realistic data on the NH_3_ concentrations that calves are exposed to, which are needed for decisions to improve animal welfare [37,38,39,40]. Regarding animal health, the interpretation of climate data obtained by point measurements has been shown to be challenging [32]. Nevertheless, high NH_3_ concentrations have been associated with respiratory health problems in cattle and calves [21,22,30,49]. The new possibility of continuous and close-proximity NH_3_ measurements near calves’ noses should allow for further investigation of associations between NH_3_ concentrations and animal health. So far, this has been difficult to achieve with methods that rely on short-term and punctual measurements of NH_3_ concentration in calf barns [32].

Previous studies showed that calves spend majority of their time lying down [50,51]. NH_3_ is primarily emitted from the floor/litter mat, where manure and urine are accumulated; therefore, we expect peak values to occur there [24,52]. We hypothesize that the high peak NH_3_ concentrations observed in the mobile measurements are caused by calves lying on the straw mat. A possible explanation for the high peak values would be that, during resting on the side of the measuring device, the mobile sensor was directly exposed to the litter mat where NH_3_ is produced. In the present study, we did not document the behavior or activity of the calves, as our primary aim was to implement the measurement method and test it for its practical application.

Furthermore, this method might be useful to investigate the effect of different management methods on air quality. It allows an accurate and simultaneous assessment of the barn climate in practice; therefore, in further investigations, the efficiency of ventilation systems might be investigated. In practice, it might, for example, allow us to determine the optimal time for litter removal, due to its ability to provide accurate climate data. Appropriate management could lead to optimizations in the barn climate and, in turn, could potentially lead to improvements in animal health. However, these questions will be further pursued in a subsequent study [53,54].

The measurement devices themselves are a limiting factor in many barn climate studies. It is difficult to find suitable measuring devices that can withstand the rough conditions in a calf barn. Among other things, major challenges for the sensor itself include the presence of high humidity, noxious gases, and dust production in the barn. Furthermore, the devices have to withstand rough conditions consecutive to animal contact, which can cause strong forces that cause damage to the sensors. In fact, sensors often corrode under normal barn climate conditions because of moisture and condensation [37]. Another negative effect is sensor drift, which might create measurement errors. A variety of factors can cause sensor drift; the environment of the sensor is important, but correct handling should also be considered [45]. To avoid measurement errors by sensor drift, the devices were regularly calibrated and used according to the manufacturer’s instructions. According to the manufacturers and external test reports, the electrochemical sensors used are specifically designed for the conditions in animal barns [37,46,47,55]. The manufacturer of the Dräger sensors indicates a two-year sensor durability [47]. For the Testo data loggers, annual inspection by the manufacturer is recommended, and no information is available for sensor durability. Some of the measurement devices used in this study are commercially available. The mobile NH_3_ sensor is not available, as it was a prototype and was only produced on a small scale; as such, it is currently not for commercial use.

Based on previous studies, we expected accumulation of NH_3_ in the microclimate; however, the magnitude of the observed peak NH_3_ concentrations was unexpected. NH_3_ concentrations measured in previous studies during different seasons in cattle and calf barns exceeded the recommended values of up to 20 ppm [48] and 25 ppm [32], but overall average NH_3_ concentrations were below the recommended limit value of 10 ppm [32,48]. In this study the average NH_3_ concentrations measured by mobile sensors was above 10 ppm; additionally, a significant portion of both stationary and mobile sensor measurements exceeded 10 ppm. Neither of the previous studies employed long-term microclimate measurements. Nevertheless, it has been suggested that high NH_3_ concentrations in combination with other non-optimal environmental conditions can be negatively associated with animal health [16,27,30]. NH_3_ concentrations of 5 ppm in combination with air pollutants resulted in increased damage to the upper respiratory tract in pigs [49]. Another study on pigs concluded that the maximal NH_3_ concentration tolerated in the air of pig housing systems is 9 ppm [56]. For calves, a recent study showed that exposition to low NH_3_ concentrations (>4 ppm) might contribute to the severity of respiratory diseases. Indeed, they observed a link between exposure to concentrations above 4 ppm and pneumonia in calves. Furthermore, they highlighted that combined measuring of barn climate parameters, such as NH_3_, temperature, and relative humidity, might provide important information on climatic clusters and the complex interaction between indoor climate and respiratory disease, which was supported by the evidence of the different climate clusters found in their study [12].

The results of the present study showed that all measured climate parameters, i.e., temperature, relative humidity, and concentrations of noxious gases, exceeded the recommended values for calf barns [20] during summer months, in some cases by large extents. High indoor air temperatures are a known problem in mechanically ventilated calf housing systems, where ventilation cannot cope with variable climate conditions over time [39]. In the present study, the overall mean value of air temperature was above 20° C in both compartments, thus constantly exceeding the recommended optimal range [20]. The high temperatures observed during the study period likely caused heat stress and negative effects on the fattened calves during the study period. Heat stress has been shown to change behavior and negatively affect feed intake and growth performance (average daily weight gain) in calves and heifers [57,58,59]. However, neither the behavior of the animals nor the daily weight gains were examined in this context in the present study. The overall mean relative humidity was above 70% and might have further contributed to heat stress. In combination with temperatures above 20 °C, the relative humidity should be between 40% and 70% [27]. Most CO_2_ concentrations remained below the recommended maximal values, although values largely in excess of the recommended value of 3000 ppm were also measured here (up to 5000 ppm, which was the upper measuring limit of the device). CO_2_ serves as an indicator for the assessment of air quality and ventilation in housing systems. If enough fresh air is delivered, inside CO_2_ concentrations should be comparable to the concentrations found in outside/fresh air conditions (about 380 ppm). CO_2_ is produced by the respiration of animals and manure decomposition [60,61,62]. The high observed CO_2_ concentrations, while not fatal, might have negative effects on calf welfare and performance. High concentrations of CO_2_ can decrease appetite; consequently, reduced daily weight gain might occur [22,63].

Values of barn climate parameters above or below the recommended thresholds are known to have a negative impact on animal health, and highly fluctuating environmental conditions also have an impact. Large fluctuations in temperature or relative humidity cause thermic stress and, thus, can have a negative impact on animal health. Consequently, this likely leads to increased mortality and morbidity rates and indirectly causes economic loss through lower daily gains and long-term survival [11]. In the analyses, positive correlations between NH_3_ concentrations measured by mobile or stationary devices and measured values of CO_2_ concentrations, temperature, and relative humidity were observed. Correlations of NH_3_ concentration with temperature and relative humidity have been described in previous studies.

Previous studies reported positive associations between NH_3_ concentration and both, indoor air temperature and relative humidity in various livestock barns, including cow barns [64] and free-stall dairy barns during different seasons [65]. Some studies reported weak correlations for point measurements of NH_3_ and other climate parameters, like CO_2_, temperature, and humidity, over different seasons [32]. Our findings align with these observations regarding CO_2_ concentrations. Air temperature and humidity significantly influence NH_3_ concentration. Our results are consistent with previous studies on diverse farm animals, such as poultry [31,38,66], sheep [26,38], pigs [40], and cattle [39,40,48,53], whereas data on calf housing are limited and, in recent studies, no such correlations could be found [12,32]. NH_3_ is released by the decomposition of urine and feces. CO_2_ release is dependent on the same decomposition process. Microorganism activity increases with increasing temperature, which might explain the positive correlations observed in our study [24,37]. Significant correlations between air temperature, relative humidity, and NH_3_ concentrations were not found in calf fattening barns in a recent study [12]. That study included a single sampling point at the feeding stand for continuous measurements of NH_3_ concentration and a measurement period of 24 h. Furthermore, punctual one-time measurements were performed. Nevertheless, the importance of combined climate monitoring to obtain relevant data on correlations with calf health was pointed out. We agree, and our results support the concept that monitoring different climate parameters is important to understand the complex interactions of barn climate and its correlations with calf health [12]. Continuous measurement methods over several weeks as used in the present study were not performed in any of the mentioned studies. Our study was conducted in one single calf fattening barn; therefore, the external validity of the findings is limited. It will be important to conduct further studies in different types of calf housing. A standardized measurement method, for example using comparable measurement devices, defined sampling intervals, and comparable measurement locations (animal level, within the compartment, directly at animals nose), may be used to confirm the observations of this study and to identify further factors that might potentially be associated with NH_3_ concentrations and other barn climate parameters.

## 5. Conclusions

This study implemented a measurement method with mobile NH_3_ sensors to measure NH_3_ concentrations in the microclimate near calves’ heads. Our findings demonstrate that NH_3_ concentrations tested with mobile sensors significantly differ from those measured by stationary sensors. Mobile sensors measured higher peak values and stronger fluctuations. The new measurement method with mobile NH_3_ sensors implemented in this study has the potential to improve future research on barn climate parameters in the microclimate range. Multipoint sampling with stationary sensors and continuous short-term measurements in the macroclimatic range did not show associations of climate parameters, such as air temperature, relative humidity, or NH_3_ concentrations with animal health indicators [32]. Long-term measurement of NH_3_ in the respiration area of the animals appears promising as a basis for management decisions on the farm. Long-term continuous measurements of other climate parameters than NH_3_ concentrations might be interesting for further research as well.

## Figures and Tables

**Table 1:** Descriptive statistical analysis of NH_3_ concentrations [ppm] measured in an experimental calf barn over the study period between June 5 and 8 August 2019.

Sensor	Overall Mean	Sd	Minimum	Maximum	n ≥ 10 ppm	% ≥ 10 ppm	n ≤ 5 ppm	% ≤ 5 ppm	n
A1	8.46	4.76	0	43.50	2924	32.78	2461	27.59	8921
A2	7.05	3.76	0	26.00	1835	20.58	3258	36.53	8918
A3	5.94	4.56	0	47.00	1856	20.82	5219	59.35	8915
A4	14.67	8.63	1	99.00	6376	72.13	521	5.89	8839
A5	13.69	11.05	0.5	96.50	5178	58.59	1241	14.04	8837
B1	7.60	5.41	0	65.50	2364	26.6	3469	39.03	8858
B2	8.78	4.63	0.5	31.00	3005	33.68	2165	24.27	8921
B3	9.40	6.49	0	45.50	3275	36.72	2531	28.37	8920
B4	11.33	8.89	0.5	98.5	3484	47.50	1475	20.11	7335
B5	13.84	9.92	1	97.5	5361	60.28	878	9.87	8894

Mean = overall mean of all individual measurements (the highest value observed during 10 min intervals was recorded) performed by the respective devices, Sd = standard deviation, A1–A5 = measurements in compartment A (see Figure 1), B1–B5 = measurements performed in compartment B; A1, A2, A3, B1, B2, B3 = stationary sensors, A4, A5, B4, B5 = mobile sensors (attached with halters to the calves’ head), n = number of measurements included in the analysis.

**Table 2:** Descriptive statistical analysis for CO_2_ concentrations [ppm] measured in an experimental calf barn over the entire study period from 5 June to 8 August 2019.

Sensor	Overall Mean	Sd	Min	Max	n ≥ 3000 ppm	% ≥ 3000 ppm	n
A1	1256	445	0	4952	90	1.02	8790
B1	1340	502	0	4966	121	1.38	8759

n = number of measurements included in the analysis, Sd = standard deviation, n > 3000 ppm = number of measured values (and proportion in %) of the respective measured values exceeding the recommended maximal value for calf barns of 3000 ppm [20]; Min = minimum measured value, Max = maximum measured value.

**Table 3:** Descriptive statistical analysis for temperature values [°C] measured in an experimental calf barn over the entire study period from 5 June to 8 August 2019.

Sensor	Overall Mean	Sd	Min	Max	n ≥ 20 °C	% ≥ 20 °C	n
A1	24.78	3.36	16.2	34.0	8154	92.07	8857
B1	24.7	3.24	15.1	33.5	8212	92.64	8864

n = number of measurements included in the analysis, Sd = standard deviation, n > 20 °C = number of measured values (and proportion in %) of the respective parameters exceeding the recommended maximal values for calf barns of 20 °C [20]; Min = minimum measured value, Max = maximum measured value.

**Table 4:** Descriptive statistical analysis for relative humidity [%rH] measured in an experimental barn over the entire study period from 5 June to 8 August 2019 for sensor A, respectively, from 5 June to 10 July 2019, for sensor B due to technical issues after that date.

Sensor	Overall Mean	+/− Sd	Min	Max	n ≥ 80% rH	% ≥ 80% rH	n ≤ 50% rH	% ≤ 50% rH	n
A1	73.2	9.11	39.5	93.9	2302	25.99	117	1.32	8857
B1	74.8	12.10	34.1	99.4	1696	34.27	128	2.59	4949

n = number of measurements included in the analysis, Sd = standard deviation, n > 80% RH, <50% RH = number of measured values (and proportion in %) of the respective parameters exceeding or falling below the recommended maximal and minimal, respectively, values for calf barns of 50–80% [20]; Min = minimum measured value, Max = maximum measured value.

## Data Availability

The data/models were deposited in an official repository. The data presented in this study are available on request from the corresponding author.

## Supplementary Materials

The following supporting information can be downloaded at: https://www.mdpi.com/article/10.3390/ani14132001/s1, Figure S1: Description DrägerSensor; File S1: R-Code Statistical Analysis.

## References

[1] Caja G., Castro-Costa A., Knight C.H. (2016). Engineering to Support Wellbeing of Dairy Animals. J. Dairy Res..

[2] Alsaaod M., Fadul M., Steiner A. (2019). Automatic Lameness Detection in Cattle. Vet. J..

[3] Vandermeulen J., Bahr C., Johnston D., Earley B., Tullo E., Fontana I., Guarino M., Exadaktylos V., Berckmans D. (2016). Early Recognition of Bovine Respiratory Disease in Calves Using Automated Continuous Monitoring of Cough Sounds. Comput. Electron. Agric..

[4] Silva F.G., Conceição C., Pereira A.M.F., Cerqueira J.L., Silva S.R. (2023). Literature Review on Technological Applications to Monitor and Evaluate Calves’ Health and Welfare. Animals.

[5] Costantino A., Fabrizio E., Calvet S. (2021). The Role of Climate Control in Monogastric Animal Farming: The Effects on Animal Welfare, Air Emissions, Productivity, Health, and Energy Use. Appl. Sci..

[6] Busato A., Steiner L., Martin S.W., Shoukri M.M., Gaillard C. (1997). Calf Health in Cow-Calf Herds in Switzerland. Prev. Vet. Med..

[7] Pardon B., Hostens M., Duchateau L., Dewulf J., Bleecker K., Deprez P. (2013). Impact of Respiratory Disease, Diarrhea, Otitis and Arthritis on Mortality and Carcass Traits in White Veal Calves. BMC Vet. Res..

[8] Van der Fels-Klerx H.J., Horst H.S., Dijkhuizen A.A. (2000). Risk Factors for Bovine Respiratory Disease in Dairy Youngstock in The Netherlands: The Perception of Experts. Livest. Prod. Sci..

[9] Kaske M., Kunz H.-J. (2007). Gesundheits- Und Haltungsmanagement in Der Kälberaufzucht. Nutztierpraxis Aktuell..

[10] Bähler C., Steiner A., Luginbühl A., Ewy A., Posthaus H., Strabel D., Kaufmann T., Regula G. (2012). Risk Factors for Death and Unwanted Early Slaughter in Swiss Veal Calves Kept at a Specific Animal Welfare Standard. Res. Vet. Sci..

[11] Roland L., Drillich M., Klein-Jöbstl D., Iwersen M. (2016). Invited Review: Influence of Climatic Conditions on the Development, Performance, and Health of Calves. J. Dairy Sci..

[12] Van Leenen K., Jouret J., Demeyer P., Van Driessche L., Cremer L., Masmeijer C., Boyen F., Deprez P., Pardon B. (2020). Associations of Barn Air Quality Parameters with Ultrasonographic Lung Lesions, Airway Inflammation and Infection in Group-Housed Calves. Prev. Vet. Med..

[13] Van Putten G., Hovi M., Garcia Trujillo R. (2000). An Ethological Definition of Animal Welfare with Special Emphasis on Pig Behaviour. Proceedings of the Second NAHWOA Workshop.

[14] Hämeenoja P. (2001). Animal Health and Welfare—Pig Production. Acta Vet. Scand..

[15] Hillman P., Gebremedhin K., Warner R. (1992). Ventilation System to Minimize Airborne Bacteria, Dust, Humidity, and Ammonia in Calf Nurseries. J. Dairy Sci..

[16] Lago A., McGuirk S.M., Bennett T.B., Cook N.B., Nordlund K.V. (2006). Calf Respiratory Disease and Pen Microenvironments in Naturally Ventilated Calf Barns in Winter. J. Dairy Sci..

[17] Van Caenegem L. (2006). Kälber Brauchen Aussenlufqualität.

[18] Caroprese M. (2008). Sheep Housing and Welfare. Small Rumin. Res..

[19] Cusack P.M.V., McMeniman N.P., Lean I.J. (2007). Feedlot Entry Characteristics and Climate: Their Relationship with Cattle Growth Rate, Bovine Respiratory Disease and Mortality. Aust. Vet. J..

[20] Bundesamt für Lebensmittelsicherheit und Veterinärwesen BLV (2009). Fachinformation Tierschutz Stallklimawerte und Ihre Messung in der Rinderhaltung.

[21] Seedorf J. (2013). Wirkung von Atmosphärischem Ammoniak Auf Nutztiere–Eine Kurzübersicht. Impact of Atmospheric Ammonia on Livestock Animals–a Minireview. Berl. Münch. Tierärztl. Wschr..

[22] Callan R.J., Garry F.B. (2002). Biosecurity and Bovine Respiratory Disease. Vet. Clin. N. Am. Food Anim. Pract..

[23] Zachary J.F., McGavin M.D. (2011). Pathologic Basis of Veterinary Disease.

[24] Atta A. (2006). Ammonia Emissions and Safety.

[25] Sevi A., Albenzio M., Muscio A., Casamassima D., Centoducati P. (2003). Effects of Litter Management on Airborne Particulates in Sheep Houses and on the Yield and Quality of Ewe Milk. Livest. Prod. Sci..

[26] García-Ramos F.J., Aguirre A.J., Barreiro P., Horcas E., Boné A., Vidal M. (2018). Applicability of Ammonia Sensors for Controlling Environmental Parameters in Accommodations for Lamb Fattening. J. Sens..

[27] International Commission of Agricultural Engineering (1984). Report of Working Group on Climatization of Animal Houses.

[28] International Commission of Agricultural Engineering (2004). Design Recommendations of Beef Cattle Housing.

[29] Wenke C., Pospiech J., Reutter T., Altmann B., Truyen U., Speck S. (2018). Impact of Different Supply Air and Recirculating Air Filtration Systems on Stable Climate, Animal Health, and Performance of Fattening Pigs in a Commercial Pig Farm. PLoS ONE.

[30] Schnyder P., Schönecker L., Schüpbach-Regula G., Meylan M. (2019). Effects of Management Practices, Animal Transport and Barn Climate on Animal Health and Antimicrobial Use in Swiss Veal Calf Operations. Prev. Vet. Med..

[31] Zheng W., Xiong Y., Gates R.S., Wang Y., Koelkebeck K.W. (2020). Air Temperature, Carbon Dioxide, and Ammonia Assessment inside a Commercial Cage Layer Barn with Manure-Drying Tunnels. Poult. Sci..

[32] Weber C., Bucher-Schnyder P., Schönecker L., Stucki D., Meylan M. (2022). Evaluation of associations between barn characteristics, results of barn climate parameter measurements and health indicators in Swiss veal calf herds. Schweiz Arch Tierheilkd.

[33] Leytem A.B., Dungan R.S., Bjorneberg D.L., Koehn A.C. (2011). Emissions of Ammonia, Methane, Carbon Dioxide, and Nitrous Oxide from Dairy Cattle Housing and Manure Management Systems. J. Environ. Qual..

[34] Camiloti T.V., Fregonesi J.A., Keyserlingk M.A.G., Weary D.M. (2012). Short Communication: Effects of Bedding Quality on the Lying Behavior of Dairy Calves. J. Dairy Sci..

[35] Hill T.M., Bateman H.G., Aldrich J.M., Quigley J.D., Schlotterbeck R.L. (2013). Short Communication: Intensive Measurements of Standing Time of Dairy Calves Housed in Individual Pens within a Naturally Ventilated, Unheated Nursery over Different Periods of the Year. J. Dairy Sci..

[36] Pedersen S.V., Di Perta E.S., Hafner S.D., Pacholski A.S., Sommer S.G. (2018). Evaluation of a Simple, Small-Plot Meteorological Technique for Measurement of Ammonia Emission: Feasibility, Costs, and Recommendations. Trans. ASABE.

[37] Ni J.-Q., Heber A.J. (2008). Sampling and Measurement of Ammonia at Animal Facilities. Adv. Agron..

[38] Zhang Y., Lisle A.T., Phillips C.J.C. (2017). Development of an Effective Sampling Strategy for Ammonia, Temperature and Relative Humidity Measurement during Sheep Transport by Ship. Biosyst. Eng..

[39] Seedorf J., Hartung J., Schröder M., Linkert K.H., Pedersen S., Takai H., Johnsen J.O., Metz J.H.M., Groot Koerkamp P.W.G., Uenk G.H. (1998). Temperature and Moisture Conditions in Livestock Buildings in Northern Europe. J. Agric. Eng. Res..

[40] Seedorf J., Hartung J. (1999). Survey of Ammonia Concentrations in Livestock Buildings. J. Agric. Sci..

[41] Schüller L.K., Heuwieser W. (2016). Measurement of Heat Stress Conditions at Cow Level and Comparison to Climate Conditions at Stationary Locations inside a Dairy Barn. J. Dairy Res..

[42] Louie A.P., Rowe J.D., Love W.J., Lehenbauer T.W., Aly S.S. (2018). Effect of the Environment on the Risk of Respiratory Disease in Preweaning Dairy Calves during Summer Months. J. Dairy Sci..

[43] GoogleMaps. Ufa AG Versuchsbetrieb. https://www.google.com/maps/place/Ufa+AG/@47.3784444,8.207364,17z/data=!3m1!4b1!4m5!3m4!1s0x4790168f2ac7c10b:0x40e5c8c62547dcdf!8m2!3d47.3784408!4d8.2095527.

[44] Coop Naturafarm (2015). Richtline Coop Naturafarm Kalb.

[45] Von Jasmund N., Schmithausen A.J., Krommweh M.S., Trimborn M., Boeker P., Büscher W. (2022). Assessment of Ammonia Sensors and Photoacoustic Measurement Systems Using a Gas Calibration Unit. Comput. Electron. Agric..

[46] Melse R.W., Ploegaert J.P.M., Ogink N.W.M. (2016). Laboratory Test of Draeger Polytron 8000 with FL-6813260 Sensor for NH3 Measurement.

[47] Nauber A., Sick M., Steiner G., Mattern-Frühwald M.-I., Mett F., Chrzan R., Sommer S. (2016). Electrochemical Gas Sensor, Liquid Electrolyte and Use of a Liquid Electrolyte. European Patent.

[48] Teye F.K., Hautala M., Pastell M., Praks J., Veermäe I., Poikalainen V., Pajumägi A., Kivinen T., Ahokas J. (2008). Microclimate and Ventilation in Estonian and Finnish Dairy Buildings. Energy Build..

[49] Hamilton T.D., Roe J.M., Webster A.J. (1996). Synergistic Role of Gaseous Ammonia in Etiology of Pasteurella Multocida-Induced Atrophic Rhinitis in Swine. J. Clin. Microbiol..

[50] Albright J.L., Stouffer D.K., Kenyon N.J., Metz J.H.M., Groenestein C.M. (1991). Behaviour of Veal Calves in Individual Stalls and Group Pens. New Trends in Veal Calf Production.

[51] Le Neidre P., Metz J.H.M., Groenestein C.M. (1991). Effects of Breed and Early Social Environment on Calf Behaviour.

[52] Jungbluth T., Hartung E., Brose G. (2001). Greenhouse Gas Emissions from Animal Houses and Manure Stores. Nutr. Cycl. Agroecosyst..

[53] Kaufman J., Linington M., Osborne V.R., Wagner-Riddle C., Wright T.C. (2015). Short Communication: Field Study of Air Ammonia Concentrations in Ontario Dairy Calf Housing Microenvironments. Can. J. Anim. Sci..

[54] Bonizzi S., Gislon G., Brasca M., Morandi S., Sandrucci A., Zucali M. (2022). Air Quality, Management Practices and Calf Health in Italian Dairy Cattle Farms. Animals.

[55] Frosch W., Bachmann K. (2016). Forschungsbericht Agrartechnik: Verfahrenstechnische Bewertungen Physikalisch-Chemischer Messprinzipien Zur Ammoniakquantifizierung in Stallanlagen Der Landwirtschaftlichen Nutztierhaltung Im Rahmen Des Projekts Zur „Modifizierung von Regeleingangsgrößen in Zwangsbelüfteten Anlagen Der Tierproduktion“.

[56] Urbain B., Gustin P., Charlier G., Coignoul F., Lambotte J.L., Grignon G., Foliguet B., Vidic B., Beerens D., Prouvost J.F. (1996). A Morphometric and Functional Study of the Toxicity of Atmospheric Ammonia in the Extrathoracic Airways in Pigs. Vet. Res. Commun..

[57] Broucek J., Kisac P., Uhrincat M. (2009). Effect of Hot Temperatures on the Hematological Parameters, Health and Performance of Calves. Int. J. Biometeorol..

[58] Bakony M., Jurkovich V. (2020). Heat Stress in Dairy Calves from Birth to Weaning. J. Dairy Res..

[59] Wang J., Li J., Wang F., Xiao J., Wang Y., Yang H., Li S., Cao Z. (2020). Heat Stress on Calves and Heifers: A Review. J. Anim. Sci. Biotechnol..

[60] Prill R. (2000). Why Measure Carbon Dioxide Inside Buildings? Extension Energy Program.

[61] Sousa P., Pedersen S. (2004). Ammonia Emission from Fattening Pig Houses in Relation to Animal Activity and Carbon Dioxide Production. Agric. Eng. Int. CIGR J..

[62] European Food Safety Authority (2009). Scientific Report on the Effects of Farming Systems on Dairy Cow Welfare and Disease. EFSA J..

[63] ASABE (2011). Manure Storage Safety.

[64] Vtoryi V., Vtoryi S., Ylyin R. Ammonia Concentration in Cow Barn under Limited Air Exchange. Proceedings of the 18th International Scientific Conference Engineering for Rural Development.

[65] Herbut P., Angrecka S. (2014). Ammonia Concentrations in a Free-Stall Dairy Barn. Ann. Anim. Sci..

[66] Weaver W.D., Meijerhof R. (1991). The Effect of Different Levels of Relative Humidity and Air Movement on Litter Conditions, Ammonia Levels, Growth, and Carcass Quality for Broiler Chickens. Poult. Sci..

