# Supplementary material for: Assessment of Ammonia Concentrations and Climatic Conditions in Calf Housing Using Stationary and Mobile Sensors
Source: Animals (Basel). 2024 Jul 7;14(13):2001. doi: 10.3390/ani14132001 (PMC11240687; doi:10.3390/ani14132001)
Supplement: Supplementary file 1 [file animals-14-02001-s001.zip › FigureS1_DescriptionDräerSensor.pdf]

Supplementary Material
Description DrägerSensor NH_3 FL

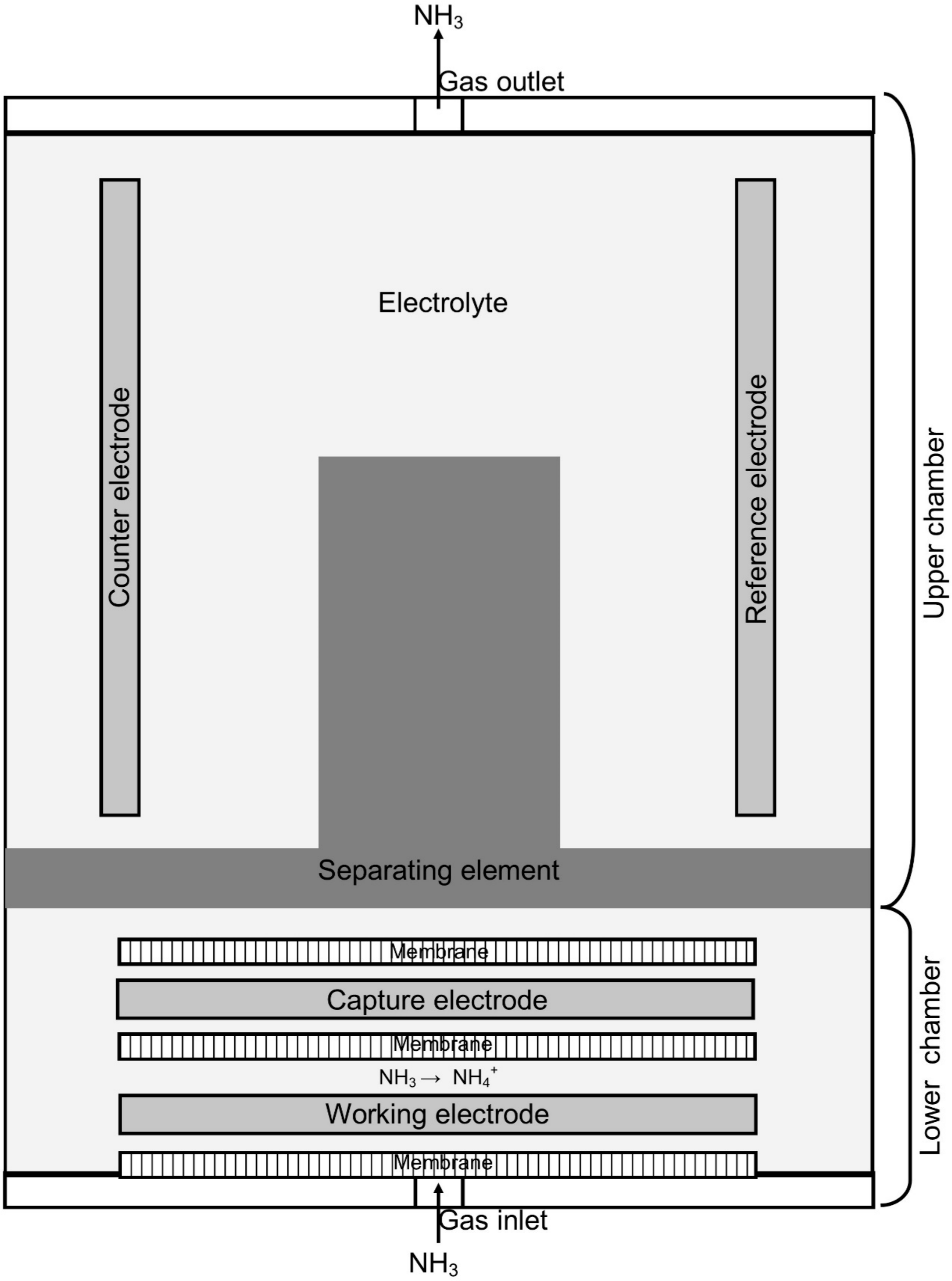

Figure S1: Schematic illustration of an electrochemical gas sensor according to [1,2].

DrägerSensor NH_3 FL Agricultural electrochemical sensor

The DrägerSensor NH_3 FL (Drägerwerk AG & Co. KGaA, 2016, Lübeck, Germany), as described by [1,2], is based on the general principle of an electrochemical sensor. An electrochemical sensos consists of working electrode and a reference electrode in contact with a liquid or solid electrolyte within an electrochemical cell. Ambient air diffuses through a membrane at the inlet, where NH_3 drives a chemical reaction generating an electrical signal. This electrical signal correlates to the NH_3 concentration in the air.

Figure S1 shows a schematic illustration of the main functional components of an electrochemical gas sensor implemented by Dräger (Dragerwerk AG & Co. KGaA, 2016, Lübeck, Germany) [1,2].

In the study of [1] and the patent [2] the Dräger electrochemical sensor (Drägerwerk AG & Co. KGaA, 2016, Lübeck, Germany) is described as follows: It consists of an electrochemical cell separated by a separating element. The electrochemical cell contains a fluid electrolyte. This electrolyte passes from the upper chamber, the electrolyte reservoir, into the lower chamber, containing the working electrode.
Ambient air enters the electrochemial cell through a membrane at the gas inlet. According to [2] electrodes in the lower chamber are protected from damage by additional membranes.
A potentiostat measures the electrode potential, defined as the voltage between the working and reference electrode. The cell current is adjusted, to maintain the electrode potential a constant level. Changes in the cell current determine the NH_3 concentrations.
Incoming NH_3 is oxidized at the working electrode. Oxidation releases electrones/ammonium ions. These ions migrate through the liquid electrolyte to the counter electrode, where they are reduced back to NH_3. An additional fourth electrode absorbs excess NH3. This electrode is called the capture electrode and avoids the uncontrolled diffusion of excess NH3 in the upper chamber of the electrochemical sensor. The NH3 produced by the reduction at the counter electrode can be released through the gas outlet opening and the sensor can be protected from overpressure.
For a more detailed description on sensor technology please refer to the patent [2] and the study of [1].

References

1. Von Jasmund, N. von; Schmithausen, A.J.; Krommweh, M.S.; Trimborn, M.; Boeker, P.; Büscher, W. Assessment of Ammonia Sensors and Photoacoustic Measurement Systems Using a Gas Calibration Unit. Computers and Electronics in Agriculture 2022, 194, doi:10.1016/j.compag.2022.106744.
2. Nauber, A.; Sick, M.; Steiner, G.; Mattern-Frühwald, M.-I.; Mett, F.; Chrzan, R.; Sommer, S. ELEKTROCHEMISCHER GASSENSOR, FLÜSSIGER ELEKTROLYT UND VERWENDUNG EINES FLÜSSIGEN ELEKTROLYTEN; ELECTROCHEMICAL GAS SENSOR, LIQUID ELECTROLYTE AND USE OF A LIQUID ELECTROLYTE (EP 3 044 576 B1) 2016.
