# Supplementary material for: Assessment of Ammonia Concentrations and Climatic Conditions in Calf Housing Using Stationary and Mobile Sensors
Source: Animals (Basel). 2024 Jul 7;14(13):2001. doi: 10.3390/ani14132001 (PMC11240687; doi:10.3390/ani14132001)

Statistical Analysis – Assessment of Ammonia Concentrations and Climatic Conditions in Calf Housing Using Stationary and Mobile Sensors

MDPI animals

Assessment of Ammonia Concentrations and Climatic Conditions in Calf Housing Using Stationary and Mobile Sensors

Julia Moser [1,2,*], Samuel Kohler [1], Jérémy Hentgen [1], Mireille Meylan [3] and Gertraud Schüpbach-Regula [2]

[1] School of Agricultural, Forest, and Food Sciences, Bern University of Applied Sciences, Länggasse 85, 3052 Zollikofen, Switzerland; samuel.kohler@bfh.ch (S.K.); jeremy.hentgen@bfh.ch (J.H.)
[2] Department of Clinical Research and Veterinary Public Health, University of Bern, Schwarzenburgstrasse 155, 3097 Liebefeld, Switzerland; gertraud.schuepbach@unibe.ch
[3] Clinic for Ruminants, Vetsuisse Faculty, University of Bern, Bremgartenstrasse 109a, 3012 Bern, Switzerland; mireille.meylan@unibe.ch
* Correspondence: julia.moser@bfh.ch; Tel.: +41-31-910-21-75

Julia Moser

2023-09-15

Packages

```
library(readxl)
library(data.table)
library(lubridate)
library(openxlsx)
library(car)
```

Load and prepare data

Three data tables with 4h-mean values of NH3 concentrations, temperature, relative humidity and CO2 concentrations were used for the statistical analysis.

```
MW_4h_mob <- read_excel("//bfhfilerbe01.bfh.ch/msj2/Desktop/Submission Paper 1/R_Analysis/MW_4h_mob.xlsx")

MW_4h_stationary <-
read_excel("//bfhfilerbe01.bfh.ch/msj2/Desktop/R_Analysis/MW_4h_stationary.xl
```

```
sx")

MW_4h_climate <- read_excel("//bfhfilerbe01.bfh.ch/msj2/Desktop/Submission
Paper 1/R_Analysis/MW_4h_climate.xlsx")

str(MW_4h_stationary)

str(MW_4h_mob)

str(MW_4h_climate)

MW_4h_stationary$Monat <- factor(MW_4h_stationary$Monat)

MW_4h_stationary$Tag <- factor(MW_4h_stationary$Tag)

MW_4h_stationary$Stunde <- factor(MW_4h_stationary$Stunde)

MW_4h_mob$Monat <- factor(MW_4h_mob$Monat)

MW_4h_mob$Tag <- factor(MW_4h_mob$Tag)

MW_4h_mob$Stunde <- factor(MW_4h_mob$Stunde)

MW_4h_climate$Monat <- factor(MW_4h_climate$Monat)

MW_4h_climate$Tag <- factor(MW_4h_climate$Tag)

MW_4h_climate$Stunde <- factor(MW_4h_climate$Stunde)

table(MW_4h_stationary$Monat,MW_4h_stationary$Tag)

table(MW_4h_mob$Monat,MW_4h_mob$Tag)
```

Merge tables

```
MW_4h_alle <-
merge(MW_4h_stationary,MW_4h_mob,by=c("Monat","Tag","Stunde"),sort=F)

str(MW_4h_alle)

View(MW_4h_alle)
```

Calculate mean values per sensor type

```
MW_4h_alle$mw_stationary <-
apply(MW_4h_alle[,c("nh3_2726","nh3_1127","nh3_1128","nh3_1135","nh3_1296","n
h3_2082")],1,mean,na.rm=T)

MW_4h_alle$mw_mob <-
apply(MW_4h_alle[,c("nh3_0448","nh3_0447","nh3_0173","nh3_0451")],1,mean,na.r
m=T)
```

Analysis mobile and stationary NH3 sensors

T-test mobile NH3 sensors and stationary NH3 sensors

```
t.test(MW_4h_alle$mw_stationary,MW_4h_alle$mw_mob,paired=T)
```

```
##
##  Paired t-test
##
## data:  MW_4h_alle$mw_stationary and MW_4h_alle$mw_mob
## t = -37.272, df = 378, p-value < 2.2e-16
## alternative hypothesis: true mean difference is not equal to 0
## 95 percent confidence interval:
##  -6.121675 -5.508145
## sample estimates:
## mean difference
##        -5.81491
```

Boxplot mobile NH3 sensors and stationary NH3 sensors

```
boxplot(MW_4h_alle$mw_stationary-MW_4h_alle$mw_mob)
```

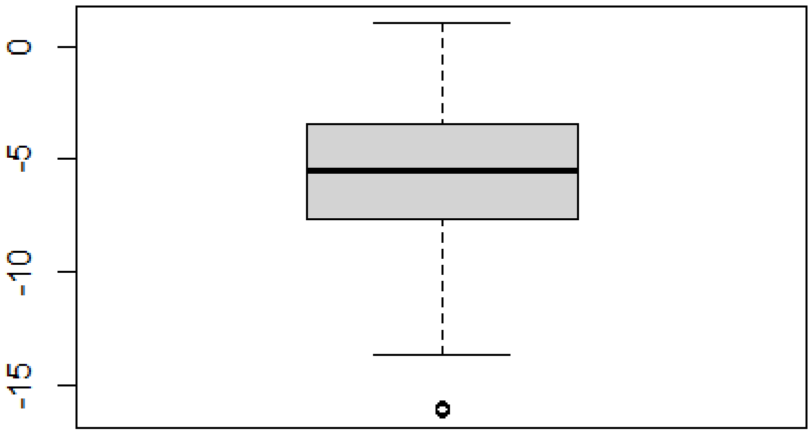

QQ-Plot mobile NH3 sensors and stationary NH3 sensors

```
qqPlot(MW_4h_alle$mw_stationary-MW_4h_alle$mw_mob)
```

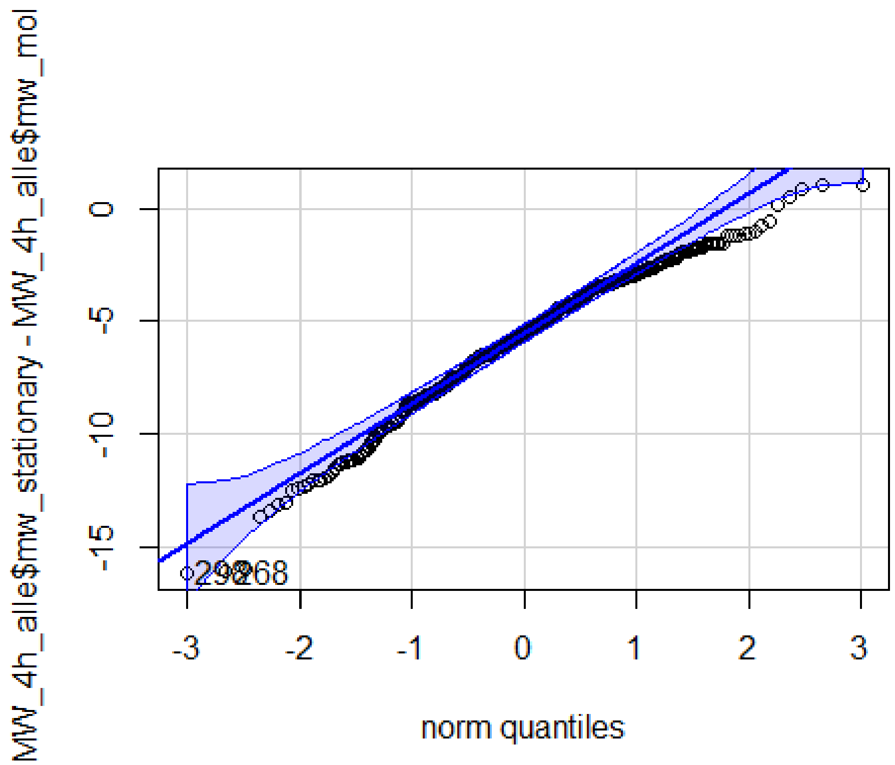


```
## [1] 298 268
```

Wilcoxon Rank Sum and Signed Rank Tests

```
wilcox.test(MW_4h_alle$mw_stationary,MW_4h_alle$mw_mob,paired=T)

##
##  Wilcoxon signed rank test with continuity correction
##
## data:  MW_4h_alle$mw_stationary and MW_4h_alle$mw_mob
## V = 23, p-value < 2.2e-16
## alternative hypothesis: true location shift is not equal to 0
```

Statistical analysis NH3 and temperature, relative humidity, CO2

Prepare data

```
MW_4h_climate <- MW_4h_climate

MW_4h_alle <-
merge(MW_4h_alle,MW_4h_climate,by=c("Monat","Tag","Stunde"),sort=F)

MW_4h_alle$mw_temp <- apply(MW_4h_alle[,c("A_temp","B_temp")],1,mean,na.rm=T)

MW_4h_alle$mw_hum <- apply(MW_4h_alle[,c("A_hum","B_hum")],1,mean,na.rm=T)

MW_4h_alle$mw_co2 <- apply(MW_4h_alle[,c("A_co2","B_co2")],1,mean,na.rm=T)
```

```
#write.xlsx(MW_4h_alle,"MW4h_alle.xlsx")
```

Data structure

```
plot(mw_stationary~mw_temp,data=MW_4h_alle)
```

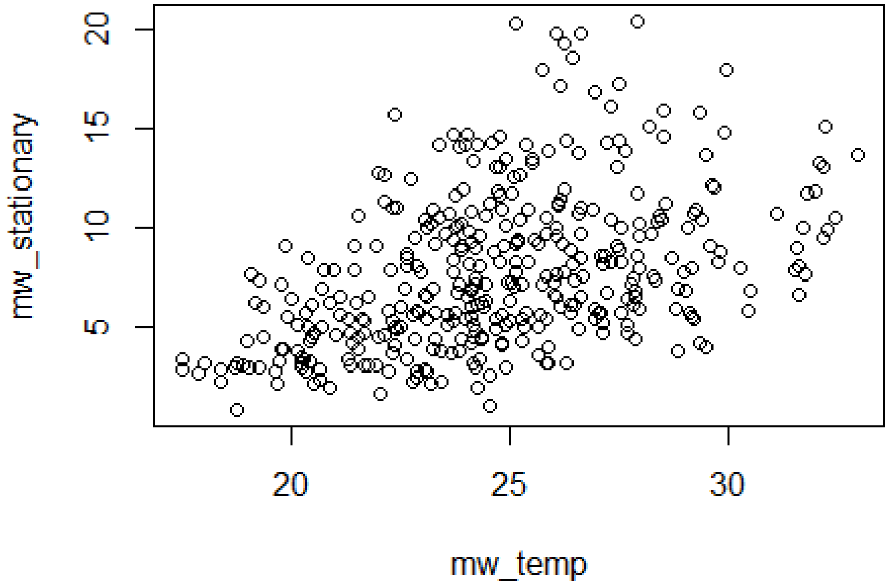


```
plot(mw_stationary~mw_hum,data=MW_4h_alle)
```

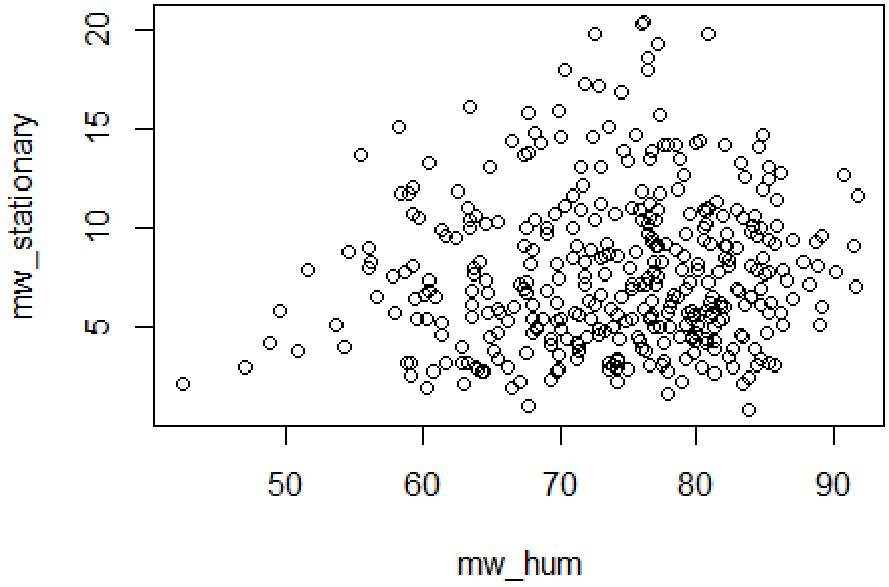


```
plot(mw_stationary~mw_co2,data=MW_4h_alle)
```

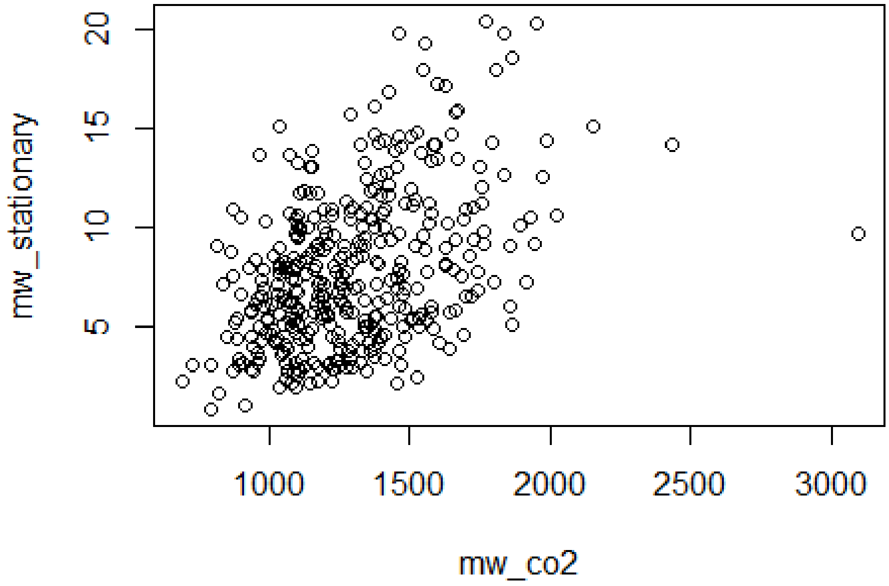

Linear models

Model 1: NH3 stationary sensors ~ temperature, humidity, and CO2 concentrations

```
m1 <- lm(mw_stationary~mw_temp+mw_hum+mw_co2,data=MW_4h_alle)

anova(m1)

## Analysis of Variance Table
##
## Response: mw_stationary
##            Df  Sum Sq Mean Sq F value    Pr(>F)
## mw_temp     1 1099.57 1099.57 131.111 < 2.2e-16 ***
## mw_hum      1  880.51  880.51 104.990 < 2.2e-16 ***
## mw_co2      1  523.00  523.00  62.362 3.192e-14 ***
## Residuals 375 3144.96    8.39
## ---
## Signif. codes:  0 '***' 0.001 '**' 0.01 '*' 0.05 '.' 0.1 ' ' 1

summary(m1)

##
## Call:
## lm(formula = mw_stationary ~ mw_temp + mw_hum + mw_co2, data = MW_4h_alle)
##
## Residuals:
##     Min      1Q  Median      3Q     Max
## -7.4016 -1.9608 -0.5123  1.4993  9.2512
##
## Coefficients:
##               Estimate Std. Error t value Pr(>|t|)
## (Intercept) -2.808e+01  2.524e+00 -11.126  < 2e-16 ***
## mw_temp      7.488e-01  5.735e-02  13.058  < 2e-16 ***
## mw_hum       1.596e-01  2.082e-02   7.664 1.56e-13 ***
## mw_co2       4.275e-03  5.413e-04   7.897 3.19e-14 ***
## ---
## Signif. codes:  0 '***' 0.001 '**' 0.01 '*' 0.05 '.' 0.1 ' ' 1
##
## Residual standard error: 2.896 on 375 degrees of freedom
## Multiple R-squared:  0.4432, Adjusted R-squared:  0.4387
## F-statistic: 99.49 on 3 and 375 DF,  p-value: < 2.2e-16

par(mfrow = (c(2,2)))
plot(m1)
```

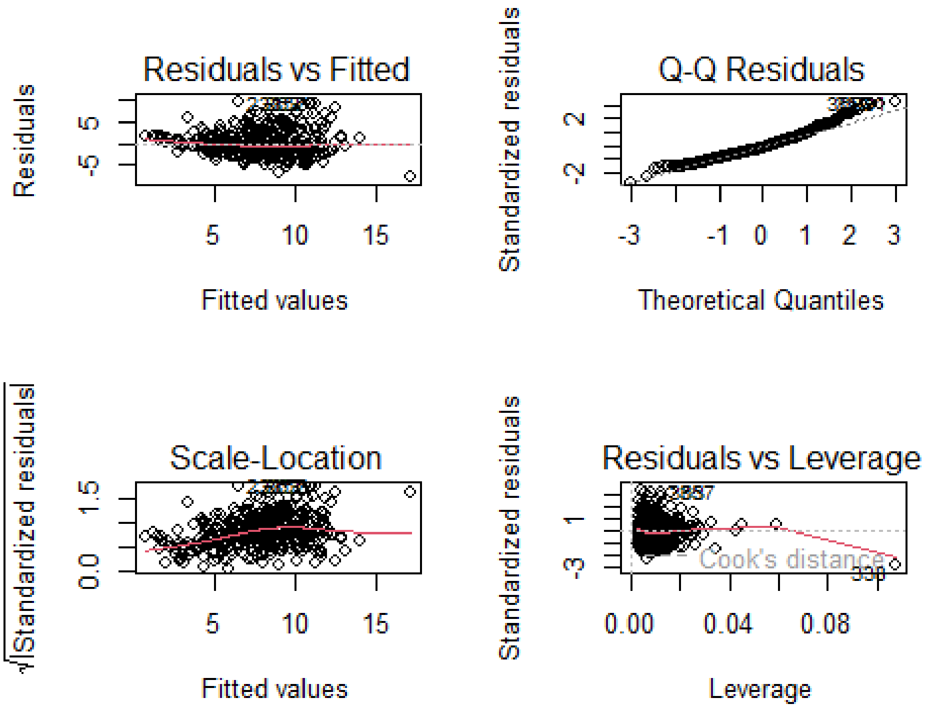

Model 2: Model NH3 stationary sensors ~ humidity and CO2 concentrations

```
m2 <- lm(mw_stationary~mw_hum+mw_co2,data=MW_4h_alle)

anova(m2)

## Analysis of Variance Table
##
## Response: mw_stationary
##            Df Sum Sq Mean Sq F value  Pr(>F)
## mw_hum      1   44.5   44.55  3.6611 0.05646 .
## mw_co2      1 1028.6 1028.57 84.5352 < 2e-16 ***
## Residuals 376 4574.9   12.17
## ---
## Signif. codes:  0 '***' 0.001 '**' 0.01 '*' 0.05 '.' 0.1 ' ' 1

summary(m2)

##
## Call:
## lm(formula = mw_stationary ~ mw_hum + mw_co2, data = MW_4h_alle)
##
## Residuals:
##     Min      1Q  Median      3Q     Max
## -8.5779 -2.6326 -0.5305  2.1760 11.0468
##
## Coefficients:
##               Estimate Std. Error t value Pr(>|t|)
```

```
## (Intercept) -0.0452005  1.5981661  -0.028    0.977
## mw_hum        0.0033965  0.0205276   0.165    0.869
## mw_co2        0.0058449  0.0006357   9.194   <2e-16 ***
## ---
## Signif. codes:  0 '***' 0.001 '**' 0.01 '*' 0.05 '.' 0.1 ' ' 1
##
## Residual standard error: 3.488 on 376 degrees of freedom
## Multiple R-squared:   0.19,  Adjusted R-squared:  0.1857
## F-statistic:  44.1 on 2 and 376 DF,  p-value: < 2.2e-16
```

```r
par(mfrow = (c(2,2)))
plot(m2)
```

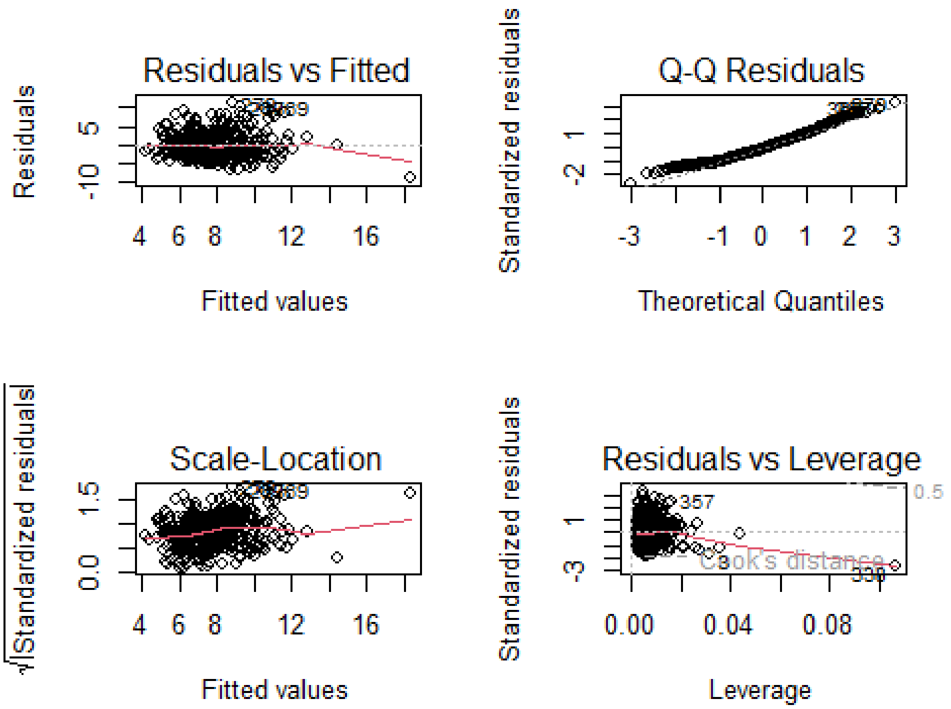

Log-transformed models

Model 3, log transformed: NH3 stationary sensors ~ temperature, humidity, and CO2 concentrations

```r
m3 <- lm(log10(mw_stationary)~mw_temp+mw_hum+mw_co2,data=MW_4h_alle)

anova(m3)
```

```
## Analysis of Variance Table
##
## Response: log10(mw_stationary)
##              Df Sum Sq Mean Sq F value    Pr(>F)
## mw_temp       1 4.7251  4.7251 180.131 < 2.2e-16 ***
```

```
## mw_hum      1 4.1117  4.1117 156.746 < 2.2e-16 ***
## mw_co2      1 1.4476  1.4476  55.187 7.469e-13 ***
## Residuals 375 9.8367  0.0262
## ---
## Signif. codes:  0 '***' 0.001 '**' 0.01 '*' 0.05 '.' 0.1 ' ' 1
```

```
summary(m3)
```

```
##
## Call:
## lm(formula = log10(mw_stationary) ~ mw_temp + mw_hum + mw_co2,
##     data = MW_4h_alle)
##
## Residuals:
##      Min       1Q   Median       3Q      Max
## -0.63809 -0.09579 -0.00919  0.12268  0.44635
##
## Coefficients:
##               Estimate Std. Error t value Pr(>|t|)
## (Intercept) -1.584e+00  1.412e-01 -11.221  < 2e-16 ***
## mw_temp      5.126e-02  3.207e-03  15.984  < 2e-16 ***
## mw_hum       1.162e-02  1.164e-03   9.978  < 2e-16 ***
## mw_co2       2.249e-04  3.027e-05   7.429 7.47e-13 ***
## ---
## Signif. codes:  0 '***' 0.001 '**' 0.01 '*' 0.05 '.' 0.1 ' ' 1
##
## Residual standard error: 0.162 on 375 degrees of freedom
## Multiple R-squared:  0.5111, Adjusted R-squared:  0.5072
## F-statistic: 130.7 on 3 and 375 DF,  p-value: < 2.2e-16
```

```
par(mfrow = (c(2,2)))
plot(m3)
```

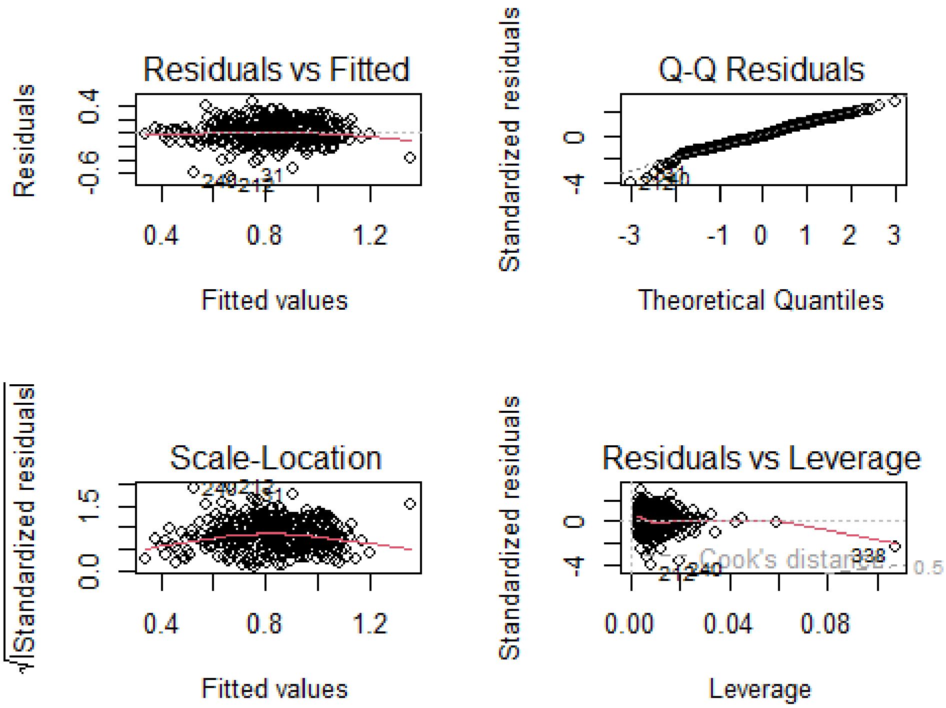


```
plot(log10(mw_stationary)~mw_temp,data=MW_4h_alle)
```

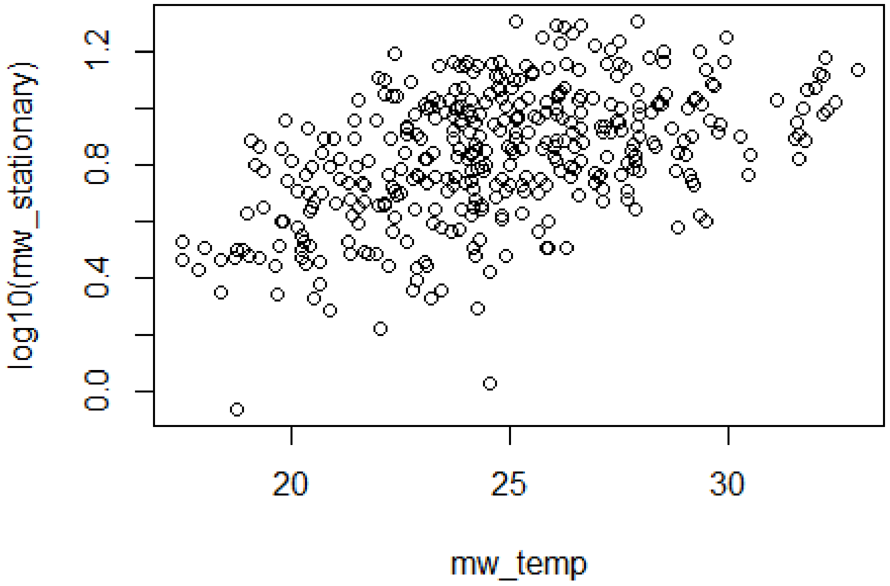


```
plot(log10(mw_stationary)~mw_hum,data=MW_4h_alle)
```

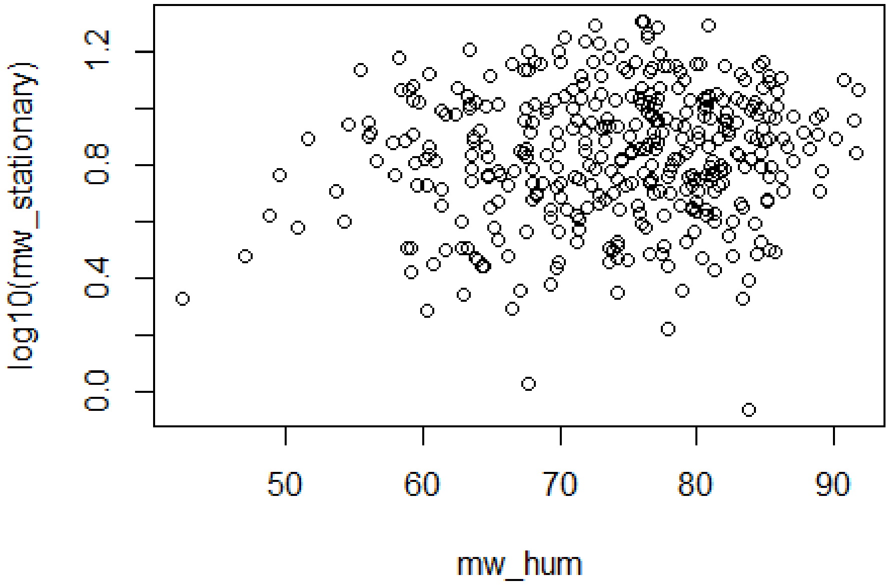


```
plot(log10(mw_stationary)~mw_co2,data=MW_4h_alle)
```

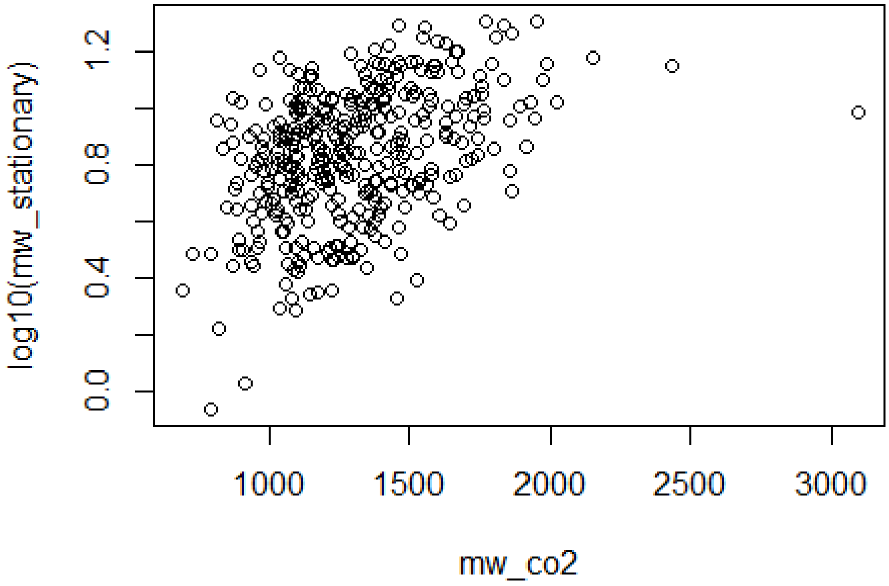


```
qqPlot(resid(m3))
```

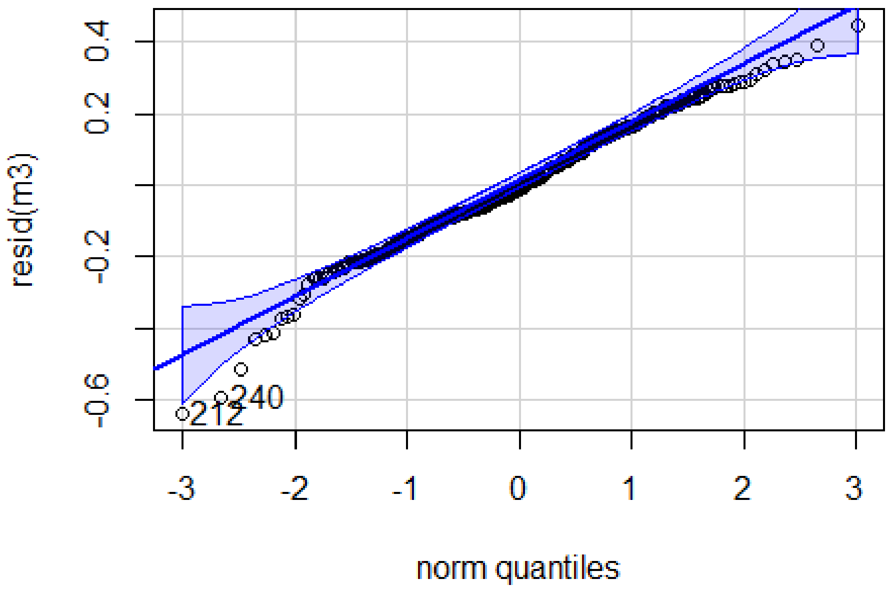


```
## [1] 212 240
```

```
plot(fitted(m3)~resid(m3))
```

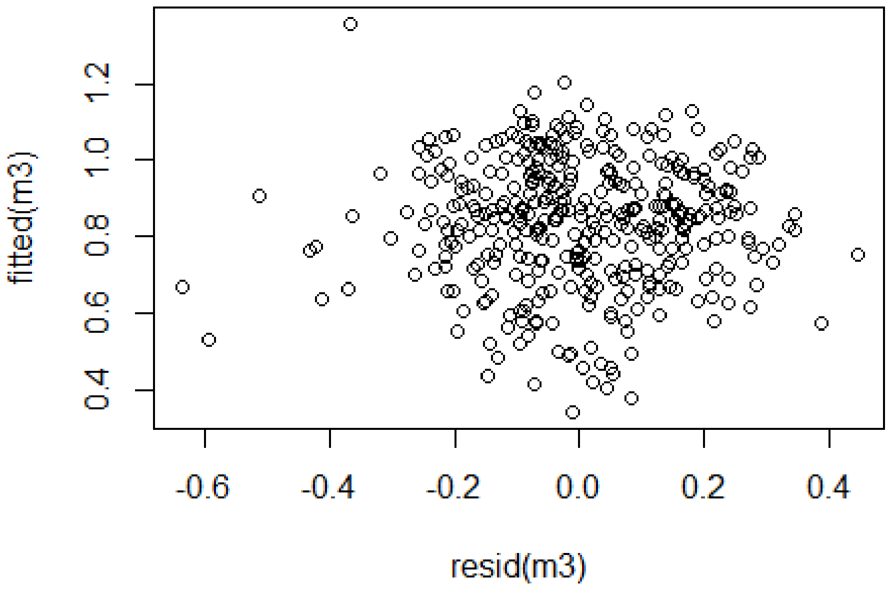

Model 4, log transformed: NH3 mobile sensors ~ temperature, humidity, and CO2 concentrations

```
m4 <- lm(log10(mw_mob)~mw_temp+mw_hum+mw_co2,data=MW_4h_alle)

anova(m4)

## Analysis of Variance Table
##
## Response: log10(mw_mob)
##            Df Sum Sq Mean Sq  F value    Pr(>F)
## mw_temp     1 3.4157  3.4157 167.9704 < 2.2e-16 ***
## mw_hum      1 1.5689  1.5689  77.1534 < 2.2e-16 ***
## mw_co2      1 0.1600  0.1600   7.8665  0.005298 **
## Residuals 375 7.6257  0.0203
## ---
## Signif. codes:  0 '***' 0.001 '**' 0.01 '*' 0.05 '.' 0.1 ' ' 1

summary(m4)

##
## Call:
## lm(formula = log10(mw_mob) ~ mw_temp + mw_hum + mw_co2, data = MW_4h_alle)
##
## Residuals:
##      Min       1Q   Median       3Q      Max
## -0.48858 -0.09624  0.01433  0.10285  0.34293
##
## Coefficients:
##               Estimate Std. Error t value Pr(>|t|)
## (Intercept) -6.024e-01  1.243e-01  -4.847 1.84e-06 ***
## mw_temp      4.134e-02  2.824e-03  14.640  < 2e-16 ***
## mw_hum       7.860e-03  1.025e-03   7.666 1.54e-13 ***
## mw_co2       7.476e-05  2.665e-05   2.805   0.0053 **
## ---
## Signif. codes:  0 '***' 0.001 '**' 0.01 '*' 0.05 '.' 0.1 ' ' 1
##
## Residual standard error: 0.1426 on 375 degrees of freedom
## Multiple R-squared:  0.4029, Adjusted R-squared:  0.3981
## F-statistic: 84.33 on 3 and 375 DF,  p-value: < 2.2e-16

par(mfrow = (c(2,2)))
plot(m4)
```

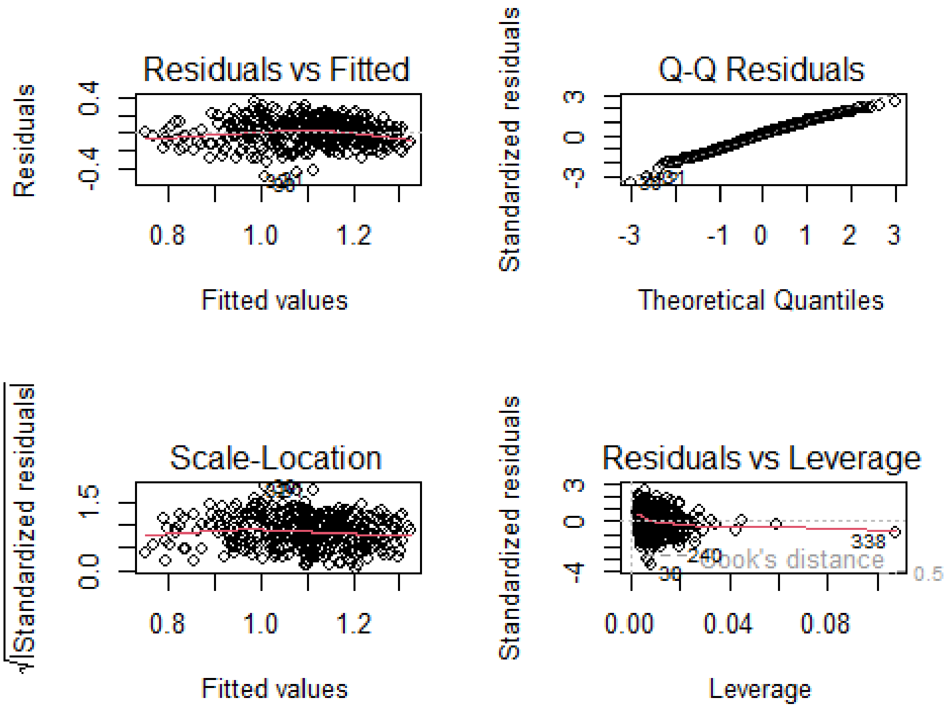


```r
plot(log10(mw_mob)~mw_temp,data=MW_4h_alle)
```

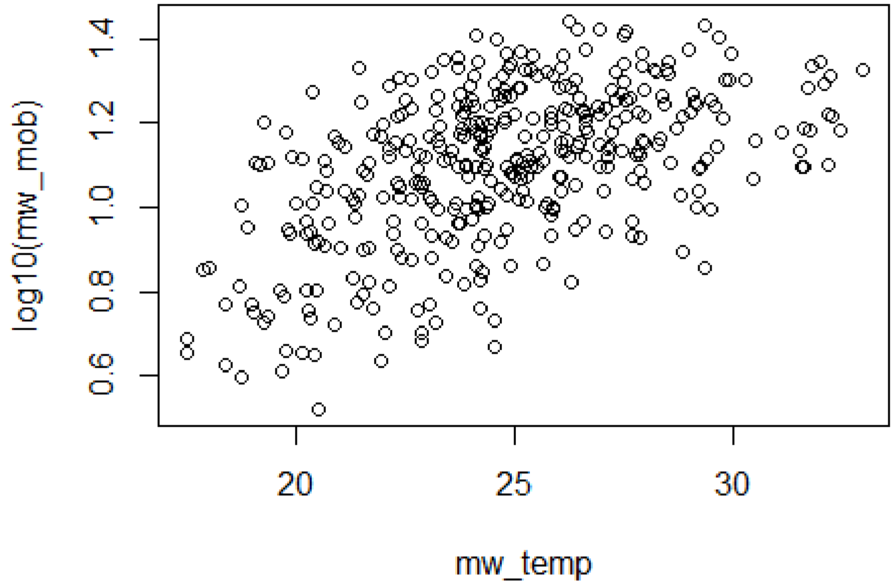


```r
plot(log10(mw_mob)~mw_hum,data=MW_4h_alle)
```

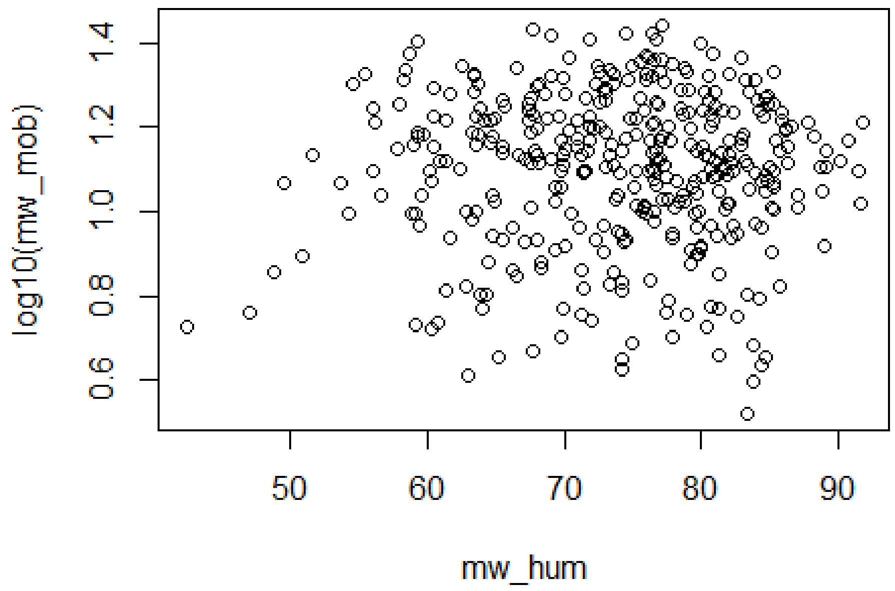


```r
plot(log10(mw_mob)~mw_co2,data=MW_4h_alle)
```

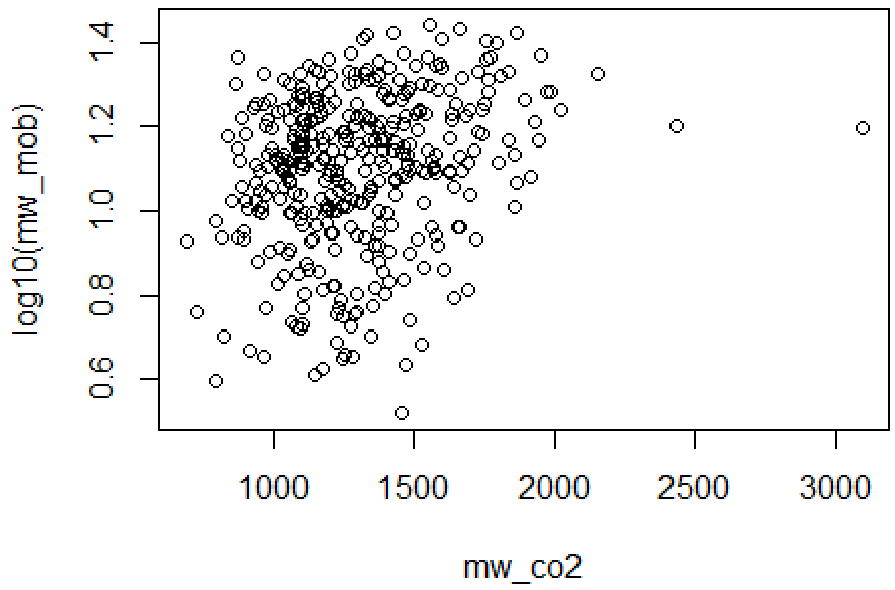


```r
qqPlot(resid(m4))
```

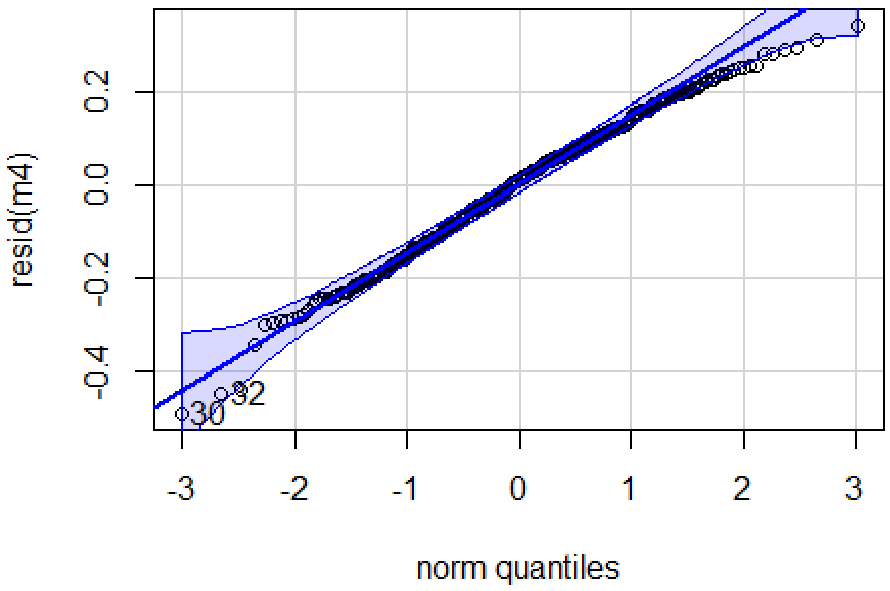


```
## [1] 30 32
```

```r
plot(fitted(m4)~resid(m4))
```

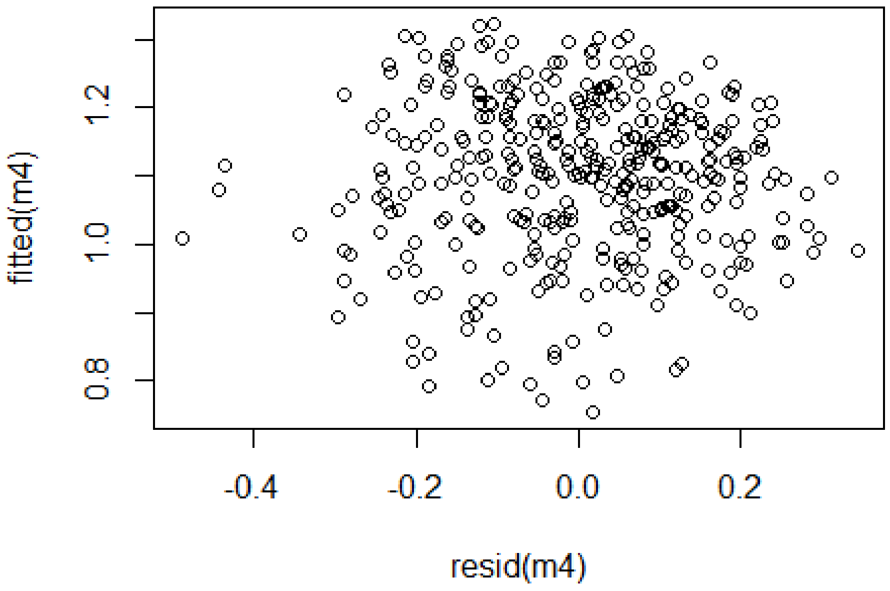

Supplement: Supplementary file 1 [file animals-14-02001-s001.zip › FileS1_R-CodeStatisticalAnalysis.pdf]
